# Communications Among Neurocytes in Parkinson's Disease Regulated by Differential Metabolism and Blood‐Brain Barrier Traversing of Chiral Gold Cluster‐MOF Integrated Nanoparticles

**DOI:** 10.1002/advs.202500026

**Published:** 2025-05-14

**Authors:** Junyang Chen, Gaoxiang Xu, Runpu Shen, Jianzhong Xu, Congcong Lu, Xin Li, Qi Feng, Qing Li

**Affiliations:** ^1^ School of Life Sciences Zhengzhou University Zhengzhou 450001 China; ^2^ Department of Orthopedics The First Affiliated Hospital of Zhengzhou University Zhengzhou 450052 China; ^3^ Zhejiang Engineering Research Center of Fat‐soluble Vitamin College of Chemistry and Chemical Engineering Shaoxing University Shaoxing 312000 China; ^4^ Department of Neurology The Second Affiliated Hospital of Zhengzhou University Zhengzhou University Zhengzhou 450052 China; ^5^ Department of Nephrology The First Affiliated Hospital of Zhengzhou University Zhengzhou 450052 China

**Keywords:** energy metabolic reprogramming, neuroinflammation, Parkinson's disease, protein corona, “three‐body problem”, ZIF‐based chiral nanomedicines

## Abstract

This study have previously reported that ZIF‐based chiral nanomedicines achieve Parkinson's disease (PD) therapy through differential metabolism and relief of neuroinflammation. However, lack of overall chirality and anti‐inflammatory capacity of nanomedicines limit the further effective solution to the nanobiological effects research in PD. Here, it dexterously loaded chiral gold nanoclusters (AuNCs) onto the inner and outer surfaces of ZIF to achieve the purpose of simultaneously improving the overall chirality and anti‐inflammatory activity of the composite nanoparticles (NPs). There are significant differences in the composition of protein corona between different chiral NPs, which elucidates the mechanism of chiral‐mediated discrepancies in metabolism and the blood‐brain barrier (BBB) traversing. Multi‐omics and biochemical techniques further reveal that chiral NPs interfere with the chemokine axis (CX3CL1/CX3CR1)‐NF‐κB‐NLRP3 and PI3K‐AKT signaling pathways, regulate communications between neurons, neural stem cells and microglia (“the three‐body problem”), and induce anti‐inflammatory efficacy of microglia mitochondrial energy metabolic reprogramming in PD. The research uncovers the biodistribution, metabolic variances, and therapeutic mechanism of chiral NPs, providing deep insights into the nanobiological effects of chiral anti‐inflammatory nanomedicines in PD therapy for future clinical transformation.

## Graphical abstract

This study have loaded chiral gold nanoclusters onto the inner and outer surfaces of ZIF and uncovers the biodistribution, metabolic variances, and therapeutic mechanism of chiral nanoparticles, providing deep insights into the nanobiological effects of chiral anti‐inflammatory nanomedicines in PD therapy for future clinical transformation.

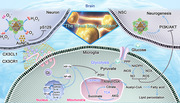

## 1 Introduction

In a system composed of three celestial bodies, the gravitational interactions between the bodies maintain the system's trajectory in a steady state. However, the influence of any one of the celestial bodies, even a minor perturbation, can result in a markedly different outcome, ultimately leading to the collapse of the three bodies, which is the “three‐body problem” postulated by Liu Cixin in “Three Bodies”.

Interestingly, a similar “three‐body problem” among nerve cells occurs in Parkinson's disease (PD), the world's second most common neurodegenerative disease.^[^ ^1^, ^2^, ^3^ ^]^ Accumulating evidence indicated that neuroinflammation is closely related to the pathogenesis of PD, and reactive oxygen species (ROS) and α‐synuclein (α‐syn) play a pivotal role in the progression of the neuroinflammatory pathology.^[^ ^4^ ^]^ Concurrently, chemotaxis‐attracted reactive microglia in the vicinity of α‐syn aggregates incite neuroinflammation and neuronal damage.^[^ ^5^ ^]^ Furthermore, ROS‐induced oxidative stress causes a shift in microglia phenotype from M2 (anti‐inflammatory) to M1 (pro‐inflammatory), resulting in the release of inflammatory mediators and contributing to the exacerbation of neuroinflammation and nerve injury.^[^ ^6^, ^7^ ^]^ It is noteworthy that ROS can further facilitate pathological factors exchanges among neuronal, astrocyte, and neural stem cell (NSCs) in the brain, thereby contributing to disease pathogenesis.^[^ ^8^, ^9^ ^]^ Additionally, the chemokine CX3CL1 and its receptor CX3CR1 regulate communication between neurons and microglia, intervening in processes such as neuroinflammation and autophagy. As a result, they modulate pathological processes including neurodegenerative diseases and pain.^[^ ^10^, ^11^ ^]^ Further, microglia and neurons can also communicate with NSCs by promoting neurogenesis, which in turn modulates pathological regression in PD.^[^ ^12^ ^]^ It was therefore previously hypothesized that microglia, neuronal cells and NSCs could form a trinity capable of synergizing neuronal cell functions through intercellular communications. Among these, the phenotypic and functional shifts of microglia caused by oxidative stress of ROS can result in overall systemic disturbances, which in turn induce the development of neuroinflammation in the progression of PD. It can therefore be proposed that the coordination of communications among neuronal cells by the blocking of pathological factors associated with ROS can result in the coordination of the “three‐body problem” among neuronal cells in neuroinflammation, thus providing a new target for the treatment of PD.

In light of the advancements in nanotechnology, nanomaterials with enzyme‐catalyzed activity are garnering significant interest as promising candidates for regulating neuroinflammation in PD. Our previous studies have found that metal‐organic frameworks (MOFs)‐based antioxidant nanoparticles (NPs) can regulate the anti‐inflammatory phenotype transformation of microglia.^[^ ^13^, ^14^, ^15^ ^]^ Therefore, we speculate whether it is possible to achieve better anti‐inflammatory effect by improving the antioxidant capacity of unit mass, to comprehensively regulate the state of microglia as well as cell communications. Regretfully, the existing MOFs‐assembled NPs strategy suffers from shortcomings that severely limit the optimization of nanomedicine anti‐inflammatory performance: 1) the number of catalysts that could be encapsulated was constrained by the dimensions of the internal space of the MOFs; and 2) the internal encapsulation impeded the contact area between the substrate and the catalysts to a certain extent. Additionally, our previous research found that chiral ZIF could achieve differential metabolism in vivo and traversing the blood‐brain barrier (BBB) in different pathways, resulting in discrepancies in brain enrichment.^[^ ^16^ ^]^ However, the preceding ZIF encapsulation strategy, which was commonly utilized, was unable to achieve the controlled and precise loading of chiral molecules, resulting in the inadequate differences of surface chirality for systematically exploring the protein corona adsorbed on the surface. Collectively, it is of the utmost importance to establish a more specific strategy that can address the deficiencies, with the goal of ultimately achieving more effective therapeutic outcomes for the exploration of biological effect mechanisms.

Encouragingly, our recently proposed novel strategy of loading gold nanoclusters (AuNCs) on the outer surface of ZIF‐8 provides an innovative way to effectively address the above deficiencies:^[^ ^17^ ^]^ 1) The controllable loading of chiral molecules on the outer side of ZIF‐8 can facilitate the precise construction of NPs for enhancing the overall chirality. As coincidental as it is noteworthy, both cysteine and glutamate, which are integral molecules in the synthesis of AuNCs, possess chiral centers. Consequently, alterations in the configuration of these chiral centers can result in changes to the overall chirality of AuNCs, specifically the emergence of L‐ and D‐forms.^[^ ^18^, ^19^ ^]^ 2) By loading the catalytic substances on the outer surface of the carriers, it can optimize the utilization of both the inner and outer space of the ZIF‐8, and enhance the loading capacity effectively; 3) Concurrently, the effective loading of the outer space can augment the contact area with the reaction substrate. Of interest, studies have revealed that AuNCs exhibit SOD and CAT‐mimetic properties.^[^ ^20^, ^21^, ^22^ ^]^ Additionally, the intrinsic fluorescence of the AuNCs may facilitate more direct tracking of the in vivo transport pathways of nanomedicines.^[^ ^23^, ^24^ ^]^

**Scheme 1:**
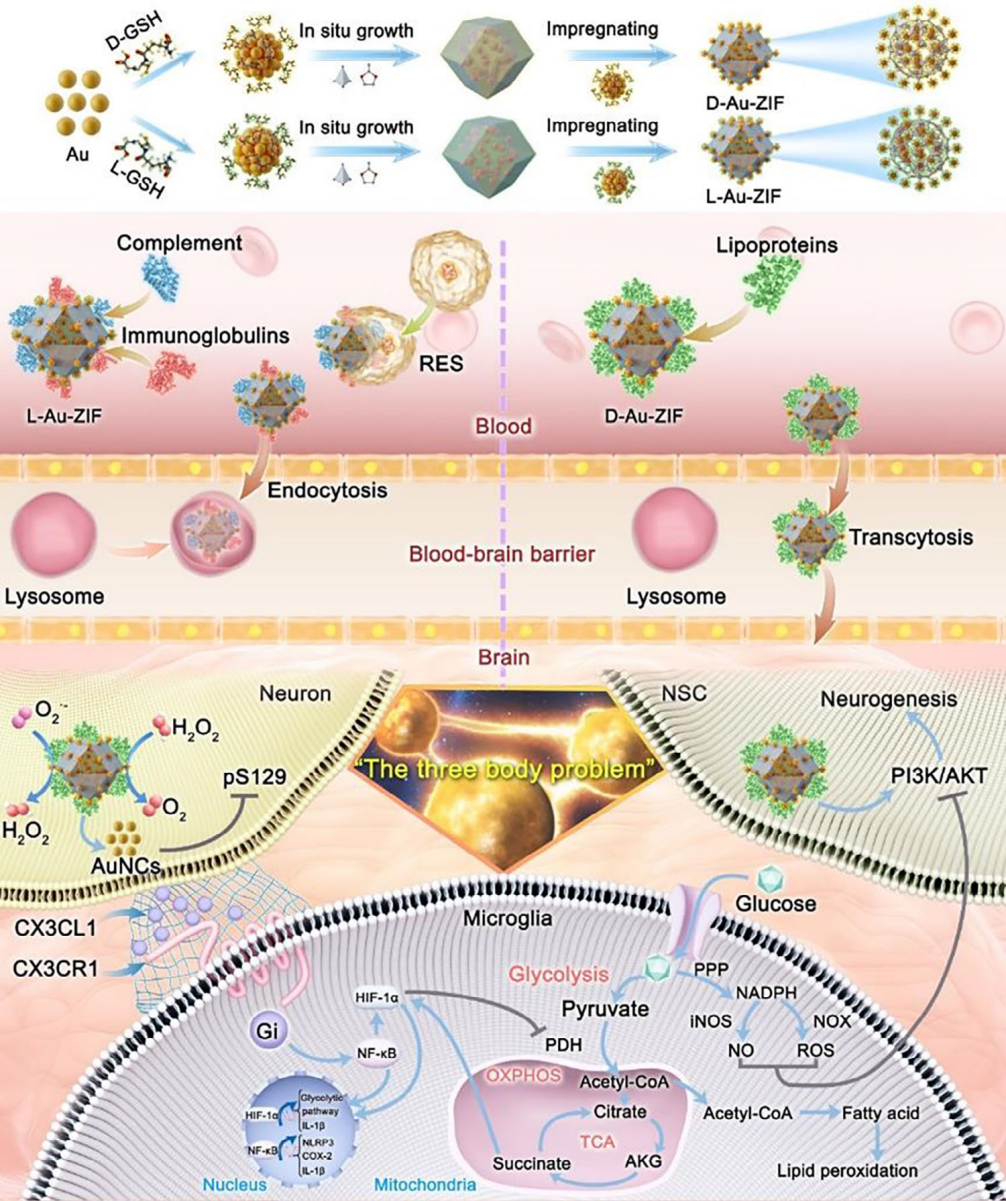
Schematic illustrating the preparation process of chiral NPs and its therapeutic mechanisms on PD based on differential metabolism, BBB traversing, and regulation of chemokine axis (CX3CL1/CX3CR1)‐mediated communications among neurons, NSCs and microglia. GSH: glutathione, RES: reticuloendothelial system, pS129: phosphorylated α‐syn, OXPHOS: oxidative phosphorylation, TCA: tricarboxylic acid, PDH: pyruvate dehydrogenase, AKG: α‐ketoglutaric acid, PPP: pentose phosphate pathway, iNOS: inducible nitric oxide synthase, NOX: nicotinamide adenine dinucleotide phosphate oxidase, NO: Nitric oxide.

The research directions are exploring the chiral‐mediated differences in in vivo metabolism and the traversing of the BBB, as well as the biological effects by increasing anti‐inflammatory efficiency per unit mass in PD treatment. The chiral structures endow NPs with different ability to adsorb proteins in the blood, thus forming protein coronas with various components on its surface, affecting their stability in the blood and potential to traverse the BBB. We selected D‐type assemblies (D‐Au‐ZIF) due to its better and desirable performance in blood stability and BBB penetration for PD treatment. In addition, through the assembly of chiral AuNCs on/in ZIF, the antioxidant activity per unit mass is notably improved, eliciting biological effect of “quantitative change causes qualitative change” in vivo to address the “three‐body problem” among neuronal cells. The research results showed that D‐Au‐ZIF could induce changes in the communications among neurons, NSCs and microglia mediated by the chemokine axis (CX3CL1/CX3CR1), thereby inducing reprogramming of mitochondrial energy metabolism, promoting neurogenesis and inhibiting neuroinflammation (**Scheme** **1**). This study aimed to improve the PD therapeutic effect of anti‐inflammatory NPs by means of chiral ligand modification and internal/external co‐assembly strategy, providing new ideas for chiral nanomedicine to mediate the energy reprogramming of microglia in anti‐inflammatory treatment for PD through differential metabolism and intervention of communications between various nerve cells.

## 2 Results and Discussion

### 2.1 Synthesis and Characterization of Chiral AuNCs and Au‐ZIF

**Figure 1:**
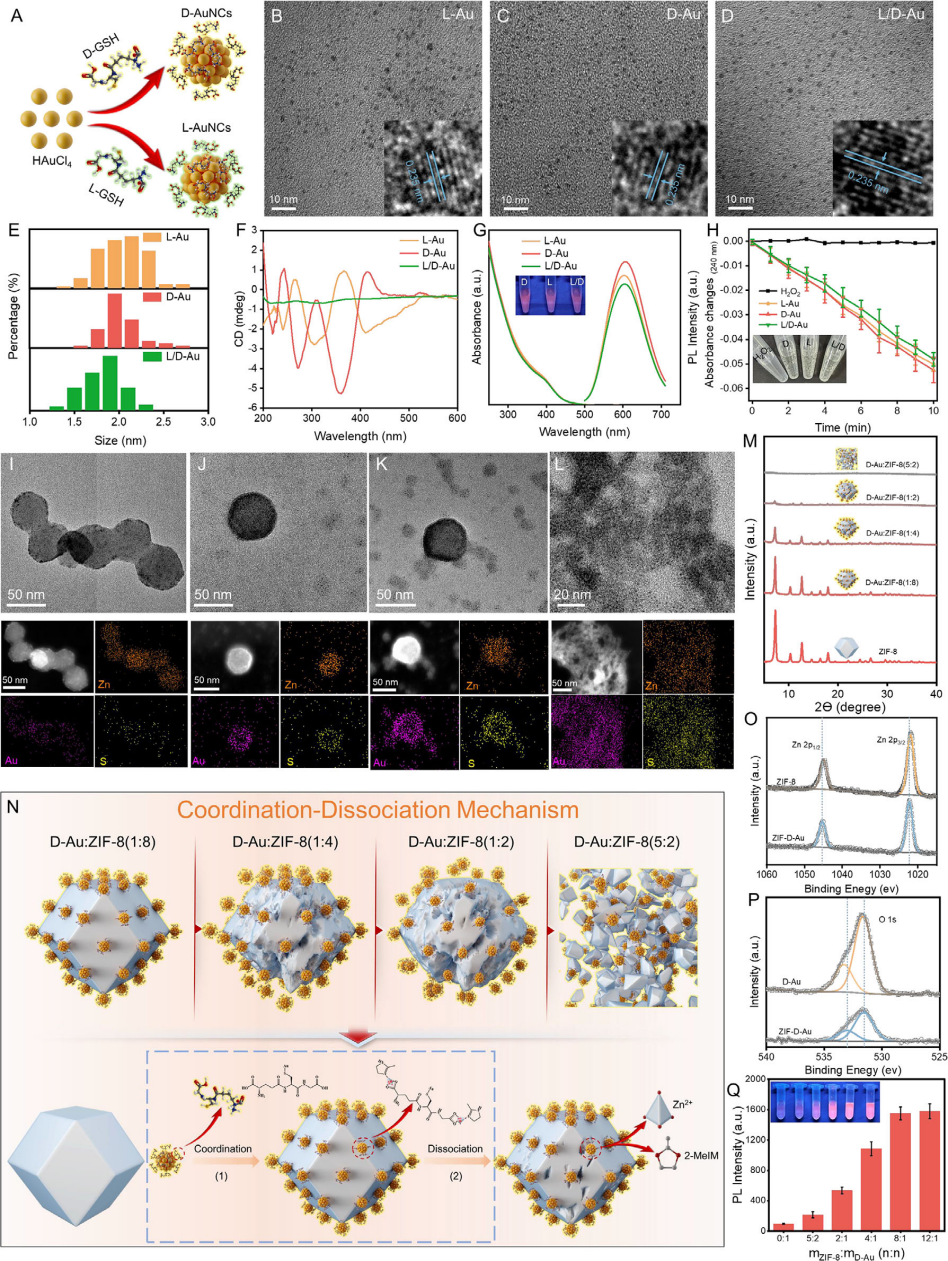
The preparation and characterization of chiral AuNCs and Au&ZIF. A) Schematic illustration of the preparation of chiral AuNCs. TEM and HERTEM images of B) L‐Au, C) D‐Au, and D) L/D‐Au. E) The size distribution of L‐Au, D‐Au, and L/D‐Au. F) CD spectra of L‐Au, D‐Au, and L/D‐Au. G) UV–vis and fluorescence spectra of L‐Au, D‐Au, and L/D‐Au. H) The CAT‐like activities of L‐Au, D‐Au, and L/D‐Au, *n* = 3. Data represent the mean ± SD. I–L) TEM images and EDS mappings of D‐Au&ZIF nanocomposites formed with the D‐Au/ZIF‐8 mass ratio of 1:8, 1:4, 1:2, and 5:2. M) XRD curves of D‐Au&ZIF nanocomposites formed with the D‐Au/ZIF‐8 mass ratio of 0:1, 1:8, 1:4, 1:2, and 5:2. N) Coordination–dissociation mechanism for the formation of the D‐Au&ZIF nanocomposites. High resolution XPS spectra of O) Zn 2p and P) O 1s. Q) Fluorescence intensity and digital photos (inset) under UV irradiation of D‐Au&ZIF at various weight ratios of ZIF‐8 to D‐Au.

GSH is a tripeptide containing three amino acids: cysteine, glutamate, and glycine. Due to the chiral centers in both cysteine and glutamate, GSH exhibits two distinct chiral enantiomeric forms: L‐GSH and D‐GSH. Consequently, the selection of the chiral form of GSH, which serves as a vital ligand in the synthesis of gold clusters (AuNCs), also results in an alteration in the overall chirality of the AuNCs, namely L‐Au and D‐Au (**Figure** **1**A). Herein, AuNCs of varying chirality were synthesized in a straightforward and gentle manner via a one‐pot hydrothermal approach, utilizing L‐/D‐GSH as the reducing agent. Transmission electron microscopy (TEM) analysis revealed that the L‐Au, D‐Au, and L/D‐Au were effectively dispersed in a spherical shape with an approximate diameter of 2 nm, indicating that the chirality of the ligands does not influence their size (Figure 1B–E; Figure S1, Supporting Information). As anticipated, the circular dichroism (CD) spectra of L‐AuNCs and D‐AuNCs exhibited a distinct CD peak in the wavelength range below 535 nm. In contrast, no notable chiral differentiation was observed for L/D‐Au, thereby confirming that the modification of diverse chiral surface ligands can influence the overall chiral attributes of the materials (Figure 1F). To elucidate the impact of the chiral properties of the materials on biodiagnostic and therapeutic applications, we conducted an in‐depth investigation into the fluorescence intensity, CAT‐like and SOD‐like activities, observing that the three AuNCs exhibited no notable discrepancy in these characteristics (Figure 1G,H; Figure S2, Supporting Information). Previously, given the ligand attraction between the zinc ions in ZIF‐8 and the carboxyl groups of glutathione in AuNCs, in addition to the electrostatic attraction between ZIF‐8 and AuNCs and the comparatively narrow pore size of ZIF‐8 relative to AuNCs, we proposed an ingenious strategy: impregnating AuNCs on the ZIF surface to generate Au&ZIF nanocomposites. It was observed that AuNCs were capable of precisely regulating the morphology of Au&ZIF composites via a “coordination‐dissociation” mechanism.^[^ ^17^ ^]^ A further area of interest is whether the strategy is affected by the chiral change of the AuNCs. As demonstrated by TEM, the AuNCs (represented by D‐AuNCs) exerted a dominant influence on the systematic evolution of Au&ZIF morphology, as evidenced by the gradual etching of the rhombic dodecahedral structure of ZIF‐8 into a spherical structure or even complete cleavage, with an increase in the mass ratio of AuNCs to ZIF‐8 from 1/8 to 5/2 (Figure 1I–L). Additionally, X‐ray diffraction (XRD) corroborated the progressive disintegration of the rhombic dodecahedral structure of Au&ZIF in accordance with the augmentation of the AuNCs/ZIF‐8 ratio (Figure 1M). Moreover, the effects of different chiral ligands on the structure of ZIF‐8 were found to be indistinguishable (Figure S3, Supporting Information).

Furthermore, we investigate whether the same “coordination‐dissociation” mechanism is operational in the assembly of chiral Au&ZIF structures. In this process, the zinc ion on the ZIF‐8 surface is coordinated with the carboxyl group of GSH in the AuNCs, leading to the rupture of the Zn─N bond and the formation of GSH‐Zn, thereby causing the disruption of the ZIF‐8 structure. Accordingly, when the mass ratio of AuNCs/ZIF‐8 is low, a reduced number of Zn─N bonds are disrupted, thereby preserving the integrity of the rhombic dodecahedral structure. Conversely, as the mass ratio of AuNCs/ZIF‐8 was augmented, a considerable number of GSH carboxyl groups were coordinated with Zn on ZIF‐8. This resulted in a substantial number of Zn─N bonds being broken and ZIF‐8 collapsing (Figure 1N). To substantiate the aforementioned conjecture, X‐ray photoelectron spectroscopy (XPS) and FTIR assays were conducted. As shown in Figure 1O,P, the Zn 2p of Au&ZIF exhibited higher binding energy compared with ZIF‐8, while the O 1s displayed a lower binding energy in comparison to AuNCs. This phenomenon can be ascribed to the decrease in the electron density of the carboxyl O‐atom following the coordination between the carboxyl of AuNCs and the zinc atoms in ZIF‐8.^[^ ^25^ ^]^ Besides, the FTIR of the GSH carboxyl group of D‐Au shifted from 1638 to 1650 cm^−1^ after assembled on ZIF‐8, which might be due to the fact that the coordination effect between the carboxyl group and zinc altered the electron cloud distribution of the carboxyl group (Figure S4, Supporting Information).^[^ ^26^ ^]^ These results confirmed that there is a coordination interaction between the carboxyl groups in the chiral gold clusters and the zinc ions on the surface of ZIF‐8. Further, it is noteworthy that the fluorescence intensity of Au&ZIF nanoparticles exhibited a tendency to increase as the ratio of AuNCs to ZIF‐8 decreased (Figure S5, Supporting Information). Upon reaching a ratio of 1:8, the assembly of ZIF structural integrity and AuNCs loading reached an optimal balance, and the loading of AuNCs was optimized to achieve the optimal fluorescence intensity and enter into the plateau period (Figure 1Q). Accordingly, the 1:8 AuNCs/ZIF‐8 ratio was also selected for further experimentation.

**Figure 2:**
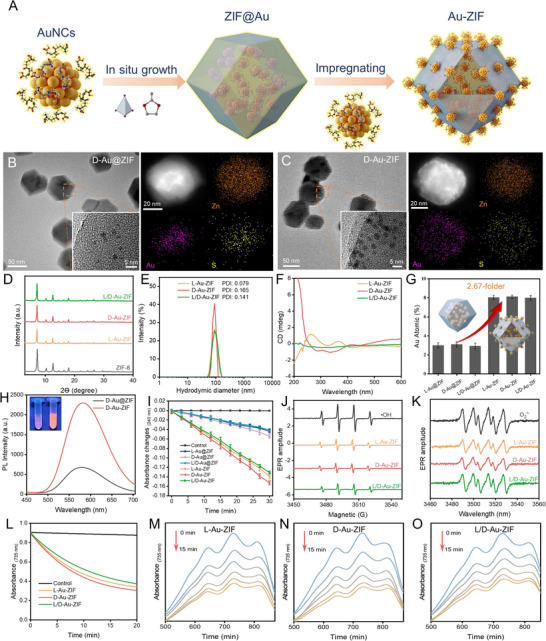
The synthesis and characterization of chiral Au‐ZIF. A) Schematic illustration of the synthesis of chiral Au‐ZIF. TEM, HERTEM, and EDS mapping images of B) D‐Au@ZIF and C) D‐Au‐ZIF. D) The XRD of ZIF‐8, L‐Au‐ZIF, D‐Au‐ZIF, and L/D‐Au‐ZIF. E) The hydrodynamic diameters with PDI value and F) CD spectra of L‐Au‐ZIF, D‐Au‐ZIF, and L/D‐Au‐ZIF. G) Au element content of corresponding nanocomposites. H) Fluorescence spectra of D‐Au@ZIF and D‐Au‐ZIF, inset displayed corresponding photos under UV irradiation. I) Decomposition of H_2_O_2_ by L‐Au@ZIF, D‐Au@ZIF, L/D‐Au@ZIF, L‐Au‐ZIF, D‐Au‐ZIF, and L/D‐Au‐ZIF, *n* = 3. Data represent the mean ± SD. J,K) The ESR spectroscopy of the reduce generation of hydroxyl radicals and superoxide radicals by L‐Au‐ZIF, D‐Au‐ZIF, and L/D‐Au‐ZIF nanocomposites. L) ABTS^•+^ spectra at 734 nm were recorded from 0 to 20 min after the addition of L‐Au‐ZIF, D‐Au‐ZIF, and L/D‐Au‐ZIF nanocomposites. M‐O) UV–vis spectra are recorded at 1–15 min after the addition of L‐Au‐ZIF, D‐Au‐ZIF, and L/D‐Au‐ZIF.

Following confirmation of the feasibility of external loading of ZIF‐8, an ingenious high‐loading strategy was constructed: First, Au@ZIF was synthesized by encapsulating AuNCs in ZIF‐8 (represented by D‐Au). Subsequently, AuNCs were assembled on the exterior of Au@ZIF, resulting in the preparation of the through‐loaded nanocomposite Au‐ZIF (**Figure** **2**A). As shown in Figure S6 (Supporting Information), once the as‐prepared Au‐ZIF supernatant solution was centrifugated, it turned transparent and nonflurescence, confirming ≈100% assembly efficiency of AuNCs on Au@ZIF surface. Further, a series of characterization assays allows for the systematic observation of the evolution of the material distribution of D‐Au in ZIF‐8 as the assembly strategy progresses (Figure 2B,C). Upon successful preparation of Au@ZIF, the rhombic dodecahedral shape of ZIF‐8 was observed to remain unchanged. High‐resolution TEM revealed the encapsulation of D‐Au into the interior of ZIF‐8, and the centrally concentrated distribution of Au elements in the EDS mappings also confirmed the successful encapsulation of D‐Au. Moreover, the rhombic dodecahedron shape of the ZIF‐8 remains unaltered following D‐Au successful assembly onto the Au@ZIF surface. This observation indicates that the internal encapsulation does not affect the “coordination‐dissociation” mechanism previously described. The successful assembly of AuNCs inside and outside of the ZIF‐8 can be seen by high‐resolution TEM, with a significantly larger loading than that achieved through the inner loading strategy alone. Furthermore, the successful synthesis of D‐Au‐ZIF is corroborated by the internal and external distribution of Au elements in EDS mappings. Likewise, the same phenomenon was observed in L‐Au‐ZIF and L/D‐Au‐ZIF (Figures S7–S10, Supporting Information). Moreover, XRD analysis demonstrated that L‐Au‐ZIF, D‐Au‐ZIF, and L/D‐Au‐ZIF retained the same morphological structure ZIF‐8, indicating that the difference in the chirality of the AuNCs did not impact the crystal structure of Au‐ZIF (Figure 2D). As anticipated, the CD spectra demonstrated that the incorporation of distinct chiral AuNCs resulted in a corresponding overall chiral transformation of Au‐ZIF (Figure 2F). The dynamic light scattering (DLS) displayed that different chiral Au‐ZIF nanoparticles exhibited similar size (≈90 nm) with low polydispersity index (PDI<0.2) and negative zeta potential, indicating their good dispersion state and high stability in biological conditions (Figure 2E; Figure S11, Supporting Information).

**Figure 3:**
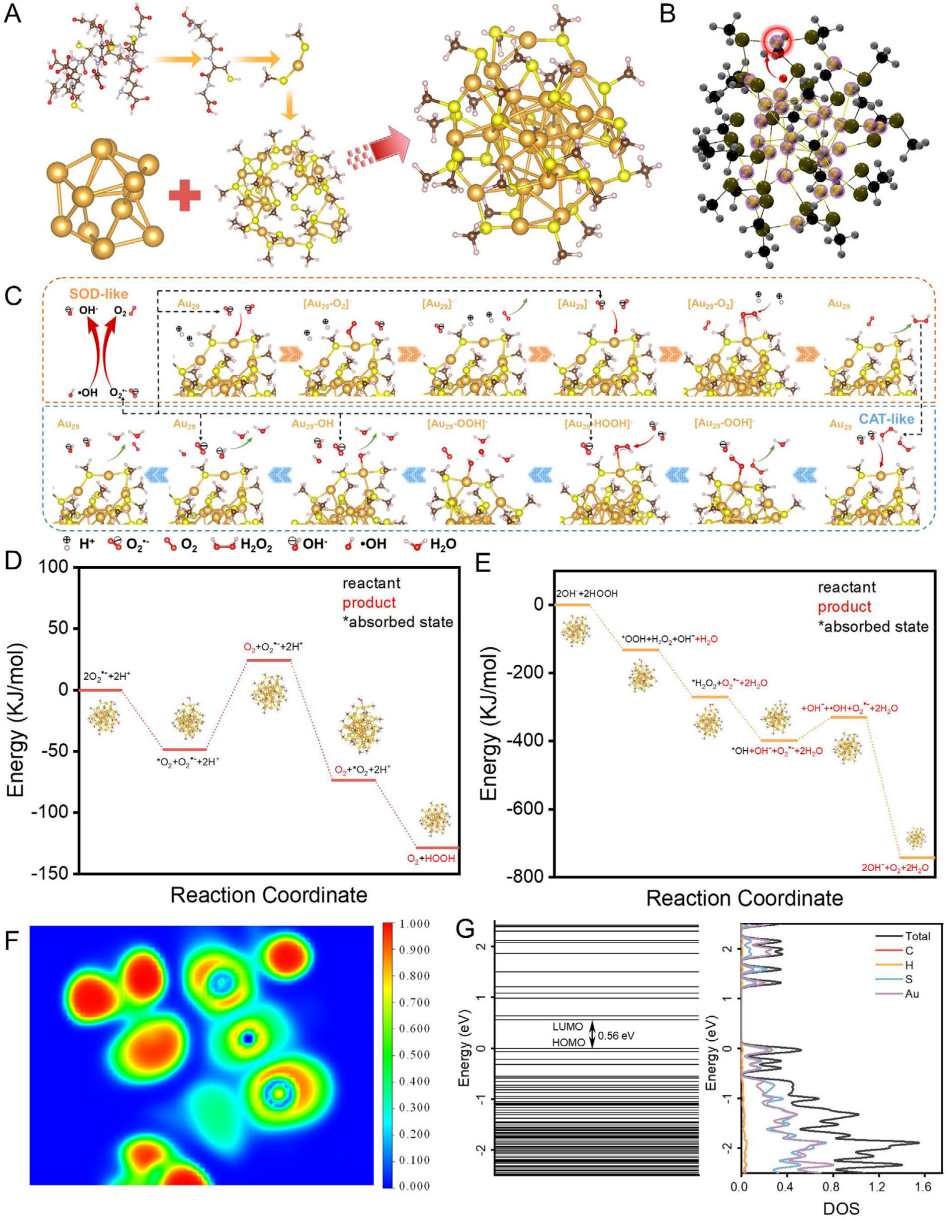
DFT calculation of AuNCs. A) Demonstration of the Au_29_ structure optimization. B) The geometrically catalytic sites optimization of AuNCs. C) Mechanism and D,E) energies profiles of SOD and CAT catalytic processes. F) The ELF images of partial Au nanoclusters. G) The energy levels of Au_29_ nanoclusters calculated by density DFT.

To elucidate the discrepancy in loading between disparate assembly methodologies, we employed inductively coupled plasma emission spectroscopy (ICP‐OES), which demonstrated that the through‐loading strategy exhibited a loading capacity that was 2.67 times greater than that of the in‐encapsulation strategy (Figure 2G). Therefore, we conclude that the through‐loading strategy has higher performance of AuNCs and take further experiments to verify it. First, fluorescence intensity, a crucial property of AuNCs, exhibits a 3.5‐fold increase relative to the internal encapsulation strategy and demonstrated the sustained stability following the implementation of the through‐loading strategy, thereby providing support for further biological experiments aimed at studying the cell‐penetrating potential of Au‐ZIF (Figure 2H; Figures S12 and S13, Supporting Information). Second, CAT‐like enzyme catalytic properties, another property of AuNCs that is most critical for the treatment of Parkinson's disease, also showed a threefold performance improvement after through‐loading compared to the internal encapsulation strategy under physiological conditions (**Figure** **3**I; Figure S15A, Supporting Information). Meanwhile, the Au‐ZIF with different chirality exhibited good and similar SOD‐like activities (Figure S14, Supporting Information). In addition, electron spin resonance (ESR) spectroscopic assays demonstrated that L‐Au‐ZIF, D‐Au‐ZIF, and L/D‐Au‐ZIF significantly scavenged the hydroxyl radicals (·OH) generated by Fenton reaction (Figure 2J) and O^2−^ produced by the xanthine‐xanthine oxidase system (Figure 2K). Besides, 2,2´‐azinobis‐(3‐ethylbenzothiazoline‐6‐sulfonate) (ABTS) free radical (ABTS^•+^) generated from the ABTS/K_2_S_2_O_8_ system was also significantly scavenged by the cascade SOD/CAT‐like activities of L‐Au‐ZIF, D‐Au‐ZIF, and L/D‐Au‐ZIF (Figure 2L–O; Figure S15B, Supporting Information). Furthermore, as shown in Figure S16 (Supporting Information), the D‐Au‐ZIF exhibits good stability under different pH or oxidative stress conditions. These results indicate that L‐Au‐ZIF, D‐Au‐ZIF, and L/D‐Au‐ZIF effectively emulate the cascade SOD/CAT functionality of the natural enzymes, thereby demonstrating considerable promise as antioxidants for mitigating PD.

### 2.2 DFT Calculations and the Mechanism of the Catalysis

Curious about the exceptional catalytic performance of Au‐ZIF, density functional theory (DFT) was employed to investigate this phenomenon. Given that the catalytic mechanism and selectivity of Au‐ZIF are primarily derived from AuNCs within the ZIF‐8 framework, the focus of analysis is primarily on AuNCs. By exploring the possible structures in the literature,^[^ ^27^ ^]^ the gold core of Au_29_ clusters protected by ligands via Au─S bond is selected for the computational model. To evaluate the catalytic behavior and the intermediate states during the chemical reactions, each ligand unit ‐SC_10_N_3_O_6_H_17_ is simplified to ‐SCH_3_ (Figure 3A). DFT optimization confirms the stability of the modeled cluster.

The catalytic sites of CAT‐like and SOD‐like enzymes were initially investigated based on localized electron affinity energy (LEAE), given that their catalysis processes were associated with the nucleophilic catalytic substrate adsorbed at the electrophilic reaction site of AuNCs. After calculation, the red site in Figure 3B is the position with the most negative electron attachment energy of AuNCs, and the purple sites are the contour of LEAE (ρ = 0.01). Considering the steric hindrance effect and the limitation of active positions on Au atoms, the gold atom labelled by red circle (Figure 3B) was selected as the catalytically active site.

Au nanoclusters have a good performance in both SOD and CAT reactions with the reaction pathways summarized in Figure 3C. The SOD reactions were involved in similar mechanisms to the general catalytic scheme of SOD reaction. The CAT reaction usually refers to the catalytic degradation of hydrogen peroxide, and the decomposition mechanism of H_2_O_2_ may involve multiple chemical stages. It is worth noting that the release of oxygen completes the CAT process, while the SOD process occurs simultaneously, and the two processes are mutually permeated. Inspired by the Arrhenius equation, we summaries the overall reaction pathways and in turn explore the transition and ground states of various types of molecules and ions. Given that the reaction efficiency is directly correlated with the activation energy between the reactants and the transition states, an investigation was conducted into the pivotal and transition states in the conversion from reactants to products. As shown in Figure 3D,E, the SOD and CAT reactions on AuNCs are energy‐declining processes, suggesting that the reactions are thermodynamically spontaneous. Besides, Figure S17 (Supporting Information) displayed that the maximum activation energy of crucial transition states is 223.7 kJ mol^−1^, which is a relatively low energy barriers, facilitating the decomposition and transformation of various ions.

To gain further insight into the catalytic mechanism, we conducted a detailed analysis of the electron distribution around the active site using the electron localization function (ELF). This approach allows us to ascertain the localization of electrons, with larger values indicating the presence of covalent bonds or lone pairs. As shown in Figure 3F and Figure S18 (Supporting Information), the ELF value of 0 indicates the absence of electron localization, whereas a value of 1 denotes the complete localization of electrons. ELF results displayed that the localization degree of electrons at the catalytic site of Au (or nearby S) is centralized with the covalent characteristics, contributing to the catalytic electron transfer process and promoting the catalyst efficiency. With the electronic structure predicted by DFT, energy levels of Au_29_ clusters were calculated. As shown in Figure 3G and Figure S19 (Supporting Information), the HOMO‐LUMO gap of the pure Au_29_ cluster is predicted to be 0.56 eV. Both of the HOMO and LUMO orbitals are assigned to Au‐S, indicating that the substrate adsorbed at Au could transfer electrons through Au‐S hybrid orbitals, which contributes to the electron transfer in the catalytic processes and further improve the catalytic efficiency. The biomimetic catalysis by AuNCs enables efficient ROS scavenging, which benefits subsequent antioxidative applications.

### 2.3 In Vitro Mitigation on Cytotoxicity Elicited by MPP^+^

**Figure 4:**
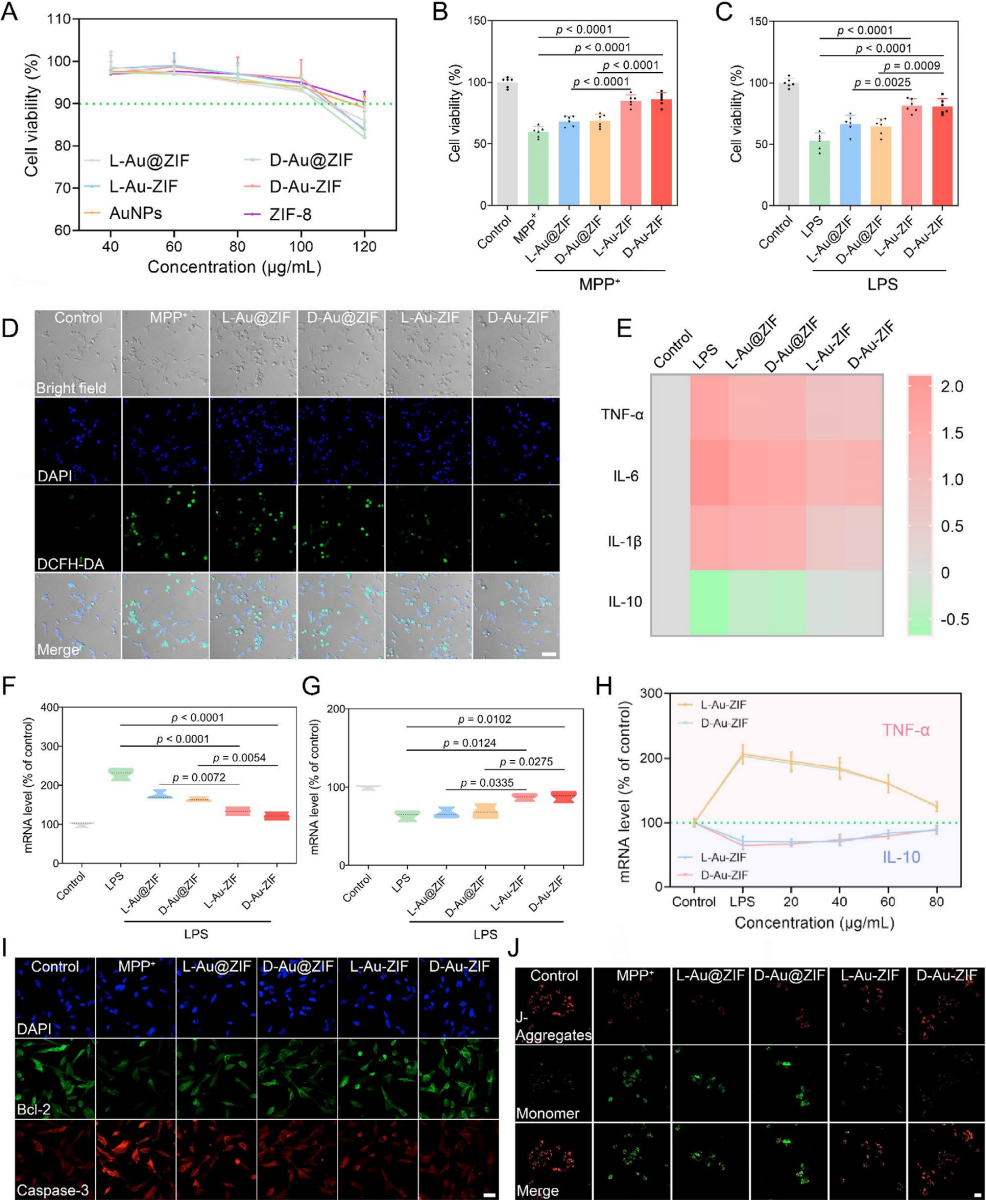
Neurocellular protection of chiral NPs in vitro. A) Cell viability of SH‐SY5Y cells exposed to NPs under various concentrations, *n* = 3. Cell viability of B) SH‐SY5Y cells and C) BV2 cells exposed to MPP^+^ or LPS following treatment with chiral NPs, *n* = 6. Data represent the mean ± SD. D) ROS levels in cells detected by fluorescent probe DCFH‐DA. The scale bar is 50 µm. E) Heatmap showing the expression levels of inflammatory factors in BV2 cells, from left to right are the representative results of control, LPS, L‐Au@ZIF, D‐Au@ZIF, L‐Au‐ZIF, and D‐Au‐ZIF groups. The expression levels of typical F) proinflammatory cytokines (TNF‐α) and G) anti‐inflammatory factors (IL‐10), *n* = 3. Data represent the mean ± SD. H) Analysis of the expression trends in inflammatory factors with L‐Au‐ZIF and D‐Au‐ZIF concentration changes, *n* = 3. Data represent the mean ± SD. I) Immunofluorescence staining of apoptosis factors in SH‐SY5Y cells. The scale bar is 20 µm. J) The mitochondrial membrane potential of SH‐SY5Y cells detected by JC‐1 probe. The scale bar is 20 µm.

First, the biocompatibility of chiral NPs on SH‐SY5Y and BV2 cells was investigated by MTT assay, in which the cell viability greater than 90% was considered biosafe with negligible cytotoxicity. The results shown in **Figure** **4**A and Figure S20A (Supporting Information) suggested that the chiral NPs exhibited no discernible cytotoxicity when the concentrations below 100 and 80 µg mL^−1^ in SH‐SY5Y and BV2 cells, respectively. Therefore, 80 µg mL^−1^ was selected as the appropriate administration concentration for subsequent in vitro experiments. Subsequently, the neuroprotective effect of chiral NPs was evaluated on the two cells lines (SH‐SY5Y and BV2) employed. As shown in Figure 4B, upon the addition of MPP^+^, SH‐SY5Y cell viability decreased to ≈60%. By contrast, the value increased to more than 80% after treatment with L‐Au‐ZIF or D‐Au‐ZIF, with more efficiacy than L‐Au@ZIF and D‐Au@ZIF. Similar tendency could also be observed in BV2 cells, in which L‐Au‐ZIF or D‐Au‐ZIF significantly rescued the decreased cell viability caused by lipopolysaccharide (LPS) stimulation (Figure 4C). In view of the antioxidant activity of chiral NPs in vitro, we then examined their effect on intracellular ROS level. As illustrated in Figure 4D and Figure S20B (Supporting Information), the ROS levels in SH‐SY5Y cells showed a notably decreasing trend after treatment with L‐Au‐ZIF or D‐Au‐ZIF, which were more significant than treating with L‐Au@ZIF or D‐Au@ZIF.

The fluctuation in ROS level will inevitably affect the cellular inflammatory response, and then take effect in the process of apoptosis and cell survival rate. Therefore, inflammatory inhibition effect of chiral NPs on microglia was evaluated. As shown in Figure 4E–G and Figure S20C,D (Supporting Information), LPS induced increased the expressions of proinflammatory factors (TNF‐α, IL‐6, IL‐1β), while inhibited the expression of anti‐inflammatory factor IL‐10. However, with the treatment of chiral NPs, the above phenomenas elicited by LPS stimulation were reversed, especially after L‐Au‐ZIF or D‐Au‐ZIF treatment. Further, with the increase of the administration concentration, neuroinflammation is gradually alleviated, confirming the inflammation remission based on L‐Au‐ZIF or D‐Au‐ZIF treatment is concentration‐dependent (Figure 4H). Subsequently, the inhibitory effect of chiral NPs on SH‐SY5Y apoptosis were detected by immunofluorescence detection, which suggested that treatment with L‐Au‐ZIF or D‐Au‐ZIF notably decreased the expression of pro‐apoptotic protein caspase‐3, while increased the expression of anti‐apoptotic protein bcl‐2 (Figure 4I; Figure S21, Supporting Information). Afterwards, the mitochondrial membrane potential was detected using JC‐1 probe. The results showed that the fluorescence ratio of JC‐1 polymer red to green decreased in MPP^+^‐treated SH‐SY5Y cells, indicating reduction in the potential of mitochondrial membrane. In contrast, both L‐Au‐ZIF and D‐Au‐ZIF treatment inhibited the decrease of mitochondrial membrane potential (Figure 4J; Figure S22, Supporting Information). Meanwhile, we found that compared with L‐Au@ZIF and D‐Au@ZIF, L‐Au‐ZIF or D‐Au‐ZIF have better effects on alleviating MPP^+^ or LPS neurotoxicity due to their higher AuNCs loading and better antioxidant activities per unit mass. Therefore, L‐Au‐ ZIF or D‐Au‐ZIF were chosen for subsequent studies.

### 2.4 The Mechanisms in Metabolism and BBB Traversing Concerning Protein Corona Formation In Vivo

**Figure 5:**
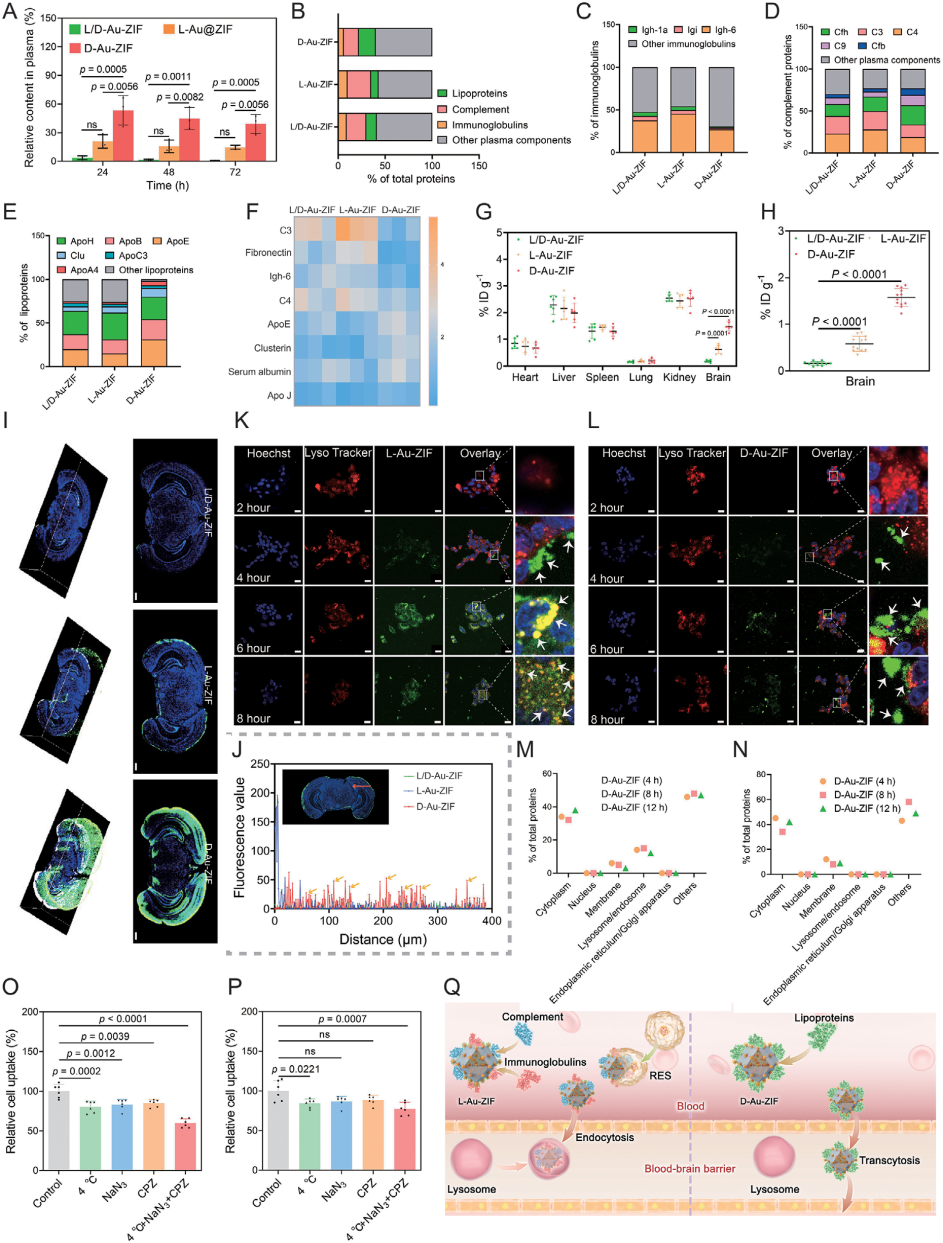
Protein corona‐mediated differences in metabolism and different BBB traversing pathways of chiral NPs. A) The plasma contents of chiral NPs at 24, 48, and 72 h after single intravenous injection, *n* = 3. Data represent the mean ± SD. LC‐MS/MS analysis of the protein coronas adsorbed by chiral NPs in blood: B) The proteins in protein corona classified by their bioinformatic and molecular functions, and the percentages of C) immunoglobulins, D) complement, E) lipoproteins, and F) heat map analysis of several representative protein coronas. Biodistribution of chiral NPs in G) major organs (*n* = 6) and H) the brain (*n* = 12) detected by ICP‐MS. Data represent the mean ± SD. I) The sections of brain tissues at the same location showing the deep penetration of chiral NPs into the brain of PD mouse models. The 3D imaging of brain sections was constructed by Image J. The scale bars are 500 µm. J) Penetration depth of chiral NPs into the brain parenchyma, as detected by the fluorescence value following the axis marked by the red arrows. Lysosome co‐localization experiment of K) L‐Au‐ZIF and L) D‐Au‐ZIF in bEnd.3 cells after co‐incubation for 2, 4, 6, and 8 h. The white arrows indicate the intracellular position of chiral NPs. The scale bars are 20 µm. Proteins adsorbed on the surface of M) L‐Au‐ZIF and N) D‐Au‐ZIF after co‐incubation of NPs with bEnd.3 cells for 4, 8, and 12 h. Internalization of O) L‐Au‐ZIF and P) D‐Au‐ZIF in bEnd.3 cells under treatment with energy‐relevant endocytosis inhibitors, *n* = 6. Data represent the mean ± SD. In control group, bEnd.3 cells were treated with chiral NPs but without endocytosis inhibitors. Q) Schematic diagram of the mechanisms by which chiral NPs‐mediated protein corona formation leads to discrepancies in their metabolism and BBB traversing avenues in vivo.

To evaluate the in vivo metabolism and brain distribution of these chiral NPs, 1‐methyl‐4‐phenyl‐1,2,3,6‐tetrahydropyridine (MPTP)‐induced PD mouse model was used. First, the blood clearance rate of chiral NPs was detected. Results shown in **Figure** **5**A indicated that compared with L/D‐Au‐ZIF and L‐Au‐ZIF, D‐Au‐ZIF showed significant more content in plasma at 24, 48 and 72 h, respectively, with negligible discrepancies between L/D‐Au‐ZIF and L‐Au‐ZIF.

After administration, the NPs may absorb serum proteins to form protein coronas, which will influence the clearance of NPs by reticuloendothelial system (RES) and immune system.^[^ ^28^ ^]^ It has been documented that the surface modification of NPs can adjust the formation of protein coronas. For example, the surface absorption of Au NPs to serum proteins could be changed by surface functionalization with lysine or cysteine.^[^ ^29^ ^]^ Meanwhile, the physicochemical properties of NPs, such as size, shape, and elasticity can also determine the patterns of protein coronas absorbed onto the NPs.^[^ ^30^ ^]^ Given the above studies, we speculate that the chirality of NPs will affect the adsorption of plasma proteins, thus eliciting the differences in its stability in the blood determined by protein corona constituents. To verify the above conjecture, the protein coronas of NPs were detected by liquid chromatography‐mass spectrometry (LC‐MS/MS). Based on bioinformatics and molecular function analysis, the results in Figure 5B and Figure S23A (Supporting Information) indicated that compared with L/D‐Au‐ZIF and L‐Au‐ZIF, D‐Au‐ZIF absorded more lipoproteins after incubating at various time. Additionally, Compared with D‐Au@ZIF, D‐Au‐ZIF also adsorbs more lipoproteins, while less immunoglobulins and complement proteins contents (Figure S23B, Supporting Information). The results in Figure 5C–E illustrated the substantial variations in protein corona components between L/D‐Au‐ZIF/L‐Au‐ZIF and D‐Au‐ZIF after incubating them in mouse blood. Subsequent heat maps summarized the representative proteins adsorbed on each group (Figure 5F), which suggested that there was increased adsorption of antiopsonin (ApoE, clusterin), as well as decreased adsorption of complement proteins C3 and C4 in D‐Au‐ZIF. In general, immunoglobulins and complement proteins are opsonin members of the protein coronas, whose content increase will lead to NPs endocytosis by macrophages, eliciting clearance of NPs by the RES system. Instead, albumin and some apolipoproteins (e.g., ApoE, ApoJ) are anti‐opsonin members that help NPs avoid endocytosis of macrophages, prolonging their half‐life of blood circulation.^[^ ^31^ ^]^ Therefore, the different half‐lives among L/D‐Au‐ZIF, L‐Au‐ZIF, and D‐Au‐ZIF could be ascribed to the conceivable reason: different chiral NPs adsorbed varied components and contents of proteins (protein coronas) in the blood, which affected their phagocytosis efficiency by RES and their blood circulation time.

Subsequently, the contents of chiral NPs in major organs were detected using inductively coupled plasma mass spectrometry (ICP‐MS). The results in Figure 5G showed that the chiral nature of NPs can alter its distribution in major organs, especially in the brain. Specifically, D‐Au‐ZIF exhibited notably higher normalized dosage accumulation (1.58% ID g^−1^) in the brain than that of L/D‐Au‐ZIF (0.16% ID g^−1^) and L‐Au‐ZIF (0.58% ID g^−1^) (Figure 5H). Although brain enrichment of nanomedicine is a prerequisite for the treatment of brain diseases, brain parenchymal penetration is also an important factor for the better therapeutic outcomes. Nanomedicine penetration within the brain parenchyma is mediated by extracellular fluid movement, pH, the presence of the extracellular matrix, and the degree of cellularity.^[^ ^32^ ^]^ In addition, some physicochemical factors including the size, surface charge, shape, and chirality of nanomedicines are also decisive factors.^[^ ^33^ ^]^ In order to investigate the effect of chirality on deep penetration of NPs into brain parenchyma, we performed brain sections and three‐dimensional stereo (3D) fluorescence imaging analysis. As shown in Figure 5I, after L/D‐Au‐ZIF treatment, fluorescence in the brain parenchyma of the brain was negligible. Meanwhile, fluorescence signal was mainly accumulated at the edge of the brain region after treatment with L‐Au‐ZIF, while following D‐Au‐ZIF treatment, fluorescence appeared in the interior of the tissues. This result can be more intuitively observed in 3D modes, which explains the difference of brain penetration between L‐Au‐ZIF and D‐Au‐ZIF. Further, we detected the penetration depth of chiral NPs. As shown in Figure 5J, after D‐Au‐ZIF treatment, the fluorescence signal was sufficiently higher at a depth of 0–400 µm. In contrast, the fluorescence of L‐Au‐ZIF was mainly concentrated in a region less than 100 µm deep from the brain periphery. These results indicated that D‐Au‐ZIF is able to penetrate deep into the brain, benefiting the accumulation of NPs in the brain.

In order to illustrated the cytological mechanisms by which chiral NPs traverse the BBB, we performed the subsequent intracellular trafficking studies within 8 h in bEnd.3 cells referred to our previous coverage.^[^ ^16^ ^]^ As shown in Figure 5K, L‐Au‐ZIF (green) appeared in the cells after 4 h, and gradually be internalized by lysosome (red) at 6 and 8 h. By contrast, only a small amount of fluorescence overlaps of D‐Au‐ZIF and lysosome could be found (Figure 5L; Figure S24, Supporting Information). Subsequent proteomic analysis after co‐incubation of NPs and bEnd.3 cells also confirmed the intracellular trafficking studies, which indicated that different from L‐Au‐ZIF (Figure 5M), D‐Au‐ZIF didn't adsorb lysosome/endosome‐related proteins during the internalization by cells (Figure 5N). These results suggested that the endocytosis process of D‐Au‐ZIF is independent of the lysosome, which is beneficial for the transcytosis and BBB traversing of NPs. Furthermore, the endocytosis modes of the chiral NPs were investigated by chemical endocytosis inhibitors. The results shown in Figure 5O,P indicated that the uptake efficiencies of both L‐Au‐ZIF and D‐Au‐ZIF were decreased by low temperature and energy‐relevant endocytosis inhibitors, demonstrating that the cell endocytosis of both chiral NPs were energy‐mediated. Overall, these results indicated that chiral NPs show significant differences in metabolism and BBB traversing in vivo. As for metabolism, chiral NPs adsorb different types of protein coronas with discrepancies in opsonins to antiopsonins ratio, making D‐Au‐ZIF advantageous in escaping RES and realizing long blood circulation. Meanwhile, the longer residence time in the bloodstream is beneficial for the enrichment of D‐Au‐ZIF in the brain. As for BBB traversing, although both chiral NPs employ energy‐dependent endocytosis in vascular endothelial cells, the endocytosis process of D‐Au‐ZIF does not pass through lysosomes, making it possible to avoid lysosomal trap and realize endotheliocyte transcytosis/BBB traversing (Figure 5Q). In view of the unsatisfactory performance of achiral NPs (L/D‐Au‐ZIF), only chiral NPs (L‐Au‐ZIF, D‐Au‐ZIF) were selected in our subsequent studies.

### 2.5 In Vivo Therapeutic Outcomes of Chiral NPs

**Figure 6:**
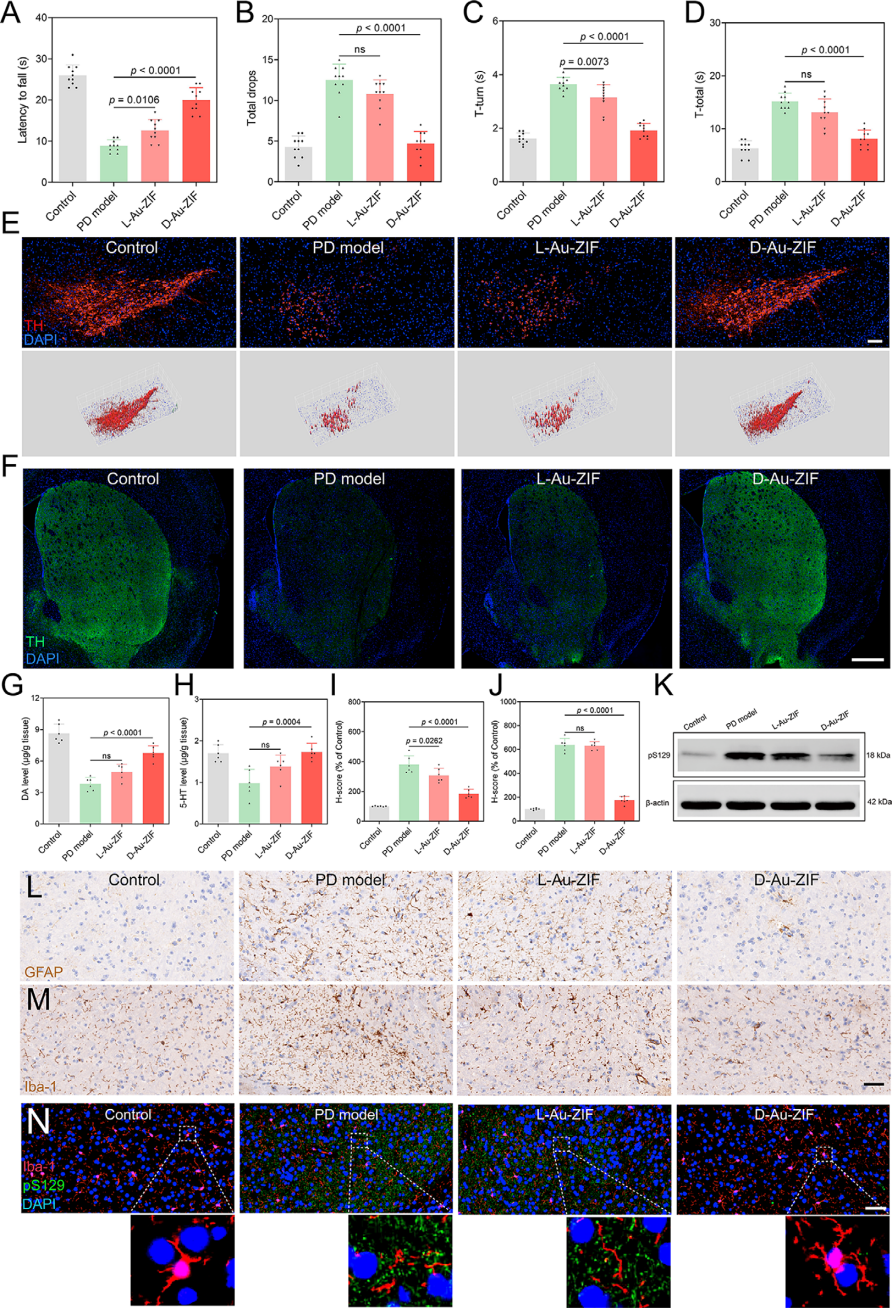
Therapeutic and inflammatory relieving outcomes of chiral NPs in PD models. The main indicators in the rotatory‐rod test including A) latency to fall and B) total drops. C) T‐turn and D) T‐total in the pole‐climbing test, *n* = 10. Data represent the mean ± SD. The expression levels of TH in E) the SNpc (scale bar: 100 µm) and F) ST region (scale bar: 500 µm) of mice brain detected by immunofluorescence staining. G) DA, and H) 5‐HT levels in the striatum of PD mice after treatment, *n* = 6. Data represent the mean ± SD. The contents of L) GFAP and M) Iba‐1 (scale bar: 50 µm) before and after treatment analyzed by immunohistochemistry and the corresponding quantitative results for I) GFAP and J) Iba‐1, *n* = 6. Data represent the mean ± SD. K) Western blotting showing the contents of pS129 after treatment. N) Co‐immunofluorescence staining of pS129 and Iba‐1 analyzed before and after treatment. The scale bar is 50 µm.

In order to assess the therapeutic outcomes of L‐Au‐ZIF and D‐Au‐ZIF on PD mouse models, we followed the operational flow outlined in Figure S25 (Supporting Information). On the fifth day after the last intravenous administration, behavioral and pathological evaluations were performed. As shown in **Figure** **6**A,B, compared with PD models, D‐Au‐ZIF‐treated mice stayed on the rotatory‐rod for a longer time with less times of drops from the rod. Further, in the pole‐climbing test, compared with PD mice, D‐Au‐ZIF‐treated PD models showed decreased values of T‐turn and T‐total (Figure 6C,D), indicating D‐Au‐ZIF improved the motor dysfunction of PD mice. Although L‐Au‐ZIF can also alleviate behavioral disorders in PD mice to a certain extent, the improvement effect is not clear and strong.

In order to illustrate the mechanisms behind the improvement in behavioral capacity, we performed pathological analyses in PD models. First, immunofluorescence assay was performed to detect the level of tyosine hudroxylase (TH), an essential enzyme in dopamine (DA) synthesis for nourishing dopaminergic neurons.^[^ ^15^ ^]^ Results shown in Figure 6E indicated that after D‐Au‐ZIF treatment, TH level in the SNpc was significantly improved compared with PD model group. Quantitative analysis of fluorescence intensity showed that TH level increased to ≈ 3 times after treatment (Figure S26A, Supporting Information). Similar results were obtained in the ST region, which showed that treatment with D‐Au‐ZIF dramatically increased TH level, and its efficiency was higher than that of L‐Au‐ZIF (Figure 6F; Figure S26B, Supporting Information). Afterwards, the levels of DA (Figure 6G), 5‐hydroxytryptamine (5‐HT) (Figure 6H), and dihydroxyphenylacetic acid (DOPAC) (Figure S26C, Supporting Information) in the ST were detected, which suggested that MPTP stimulation decreased the contents of these vital neurotransmitter, while D‐Au‐ZIF treatment rescued their levels.

Considering the superior antioxidant activity of the chiral NPs in vitro, it is reasonable to associate the improvement in PD symptoms with their antioxidant activity. Therefore, we obtained the SNpc tissue of mice and detected oxidative stress‐relevant indexes. As shown in Figure S26D,E (Supporting Information), D‐Au‐ZIF‐treated mice showed significant elevation in GSH/GSSG and SOD activity, respectively. It is worth noting that L‐Au‐ZIF could also improve GSH/GSSG and SOD activity, but its efficiency is obviously lower than D‐Au‐ZIF. Glial cells, especially microglia, play an irreplaceable role in the process of sensing external ROS pressure, and adjusting the immune inflammatory homeostasis in the central nervous system.^[^ ^16^ ^]^ Immunohistochemical and the corresponding quantitative results showed that D‐Au‐ZIF significantly alleviated the overactivation of astrocytes and microglia, which is a pathological symptom of PD caused by MPTP stimulation (Figure 6I–M). Meanwhile, the expression of the PD pathological protein, phosphorylated α‐syn (pS129), was also inhibited by treatment with D‐Au‐ZIF (Figure 6K; Figure S26F, Supporting Information). Further, considering the close relationship between microglia state, pathological protein, and neuroinflammation, we performed co‐immunofluorescence experiments of pS129 and Iba‐1 in the SNpc of mice brain. As shown in Figure 6N and Figure S26G,H (Supporting Information), the microglia in PD models showed irregular cell morphologies, while in the brains of the control group and the D‐Au‐ZIF treatment group, the cell body and synapse of microglia were intact, accompanying with decreased pS129 content, indicated the normal physiological functions of microglia.

### 2.6 Therapeutic Mechanism with Respect to Mitochondrial Energy Reprogramming Mediated by D‐Au‐ZIF in PD Model

**Figure 7:**
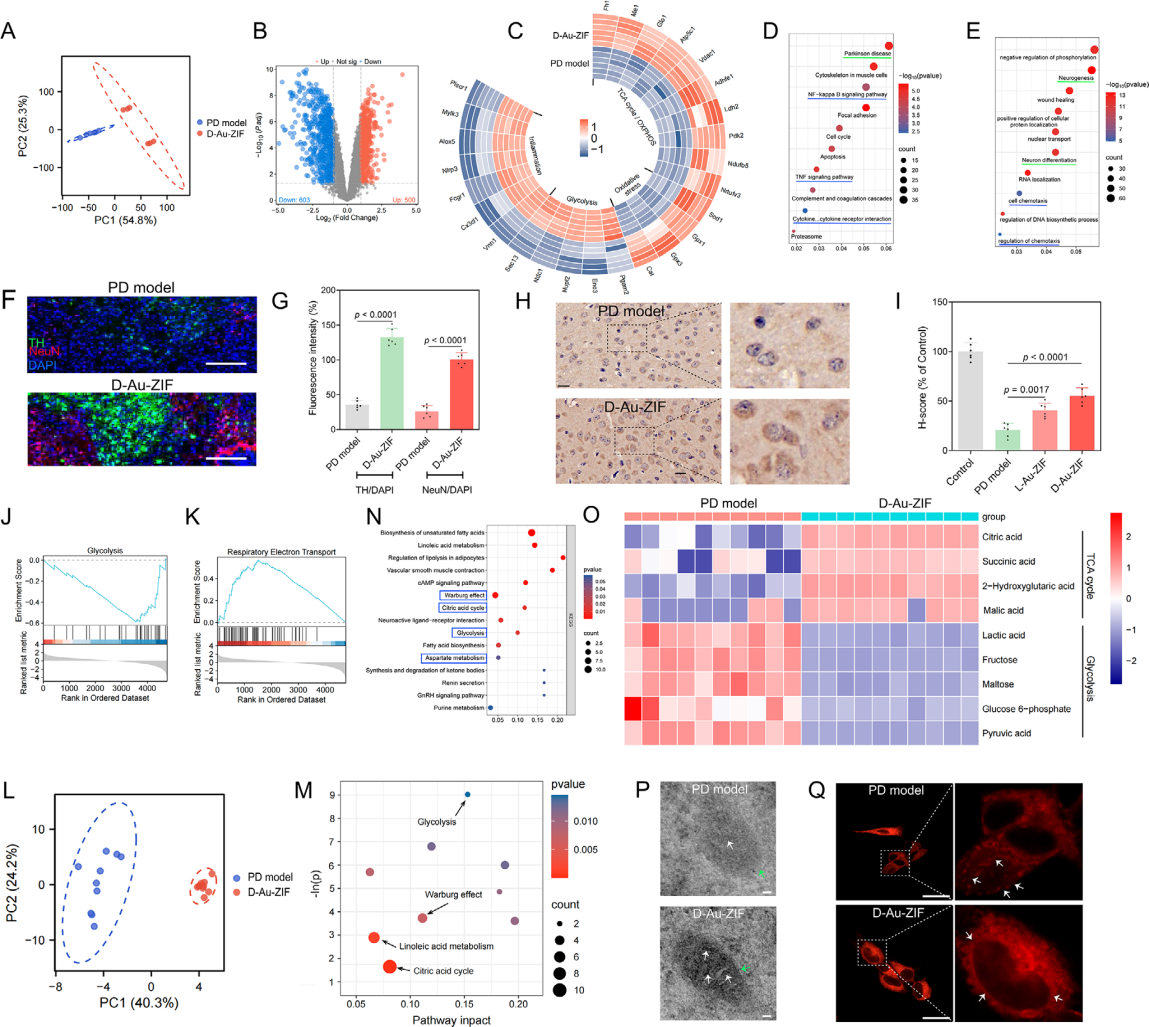
Transcriptomic and metabolomic analyses in the in vivo therapeutic mechanisms of D‐Au‐ZIF. A) PCA results of genes in PD model and D‐Au‐ZIF groups in transcriptomic analysis. B) Volcano plots results showing the DEGs after treatment with D‐Au‐ZIF. C) Circular heatmap showing DEGs related to inflammation, glycolysis, oxidative stress, and TCA cycle/OXPHOS in the mice brain after treating with D‐Au‐ZIF. D) KEGG enrichment and E) GO enrichment analyses of the identified DEGs. The green and blue underscores marked targets of interest associated with PD pathology and signaling pathways, respectively. F) Co‐immunofluorescence staining of TH and NeuN, and G) the corresponding quantitative results, for analyzing the role of D‐Au‐ZIF in promoting neurogenesis in PD models, *n* = 6. Data represent the mean ± SD. The scale bars are 200 µm. H) The expression of BrdU in brain tissue detected by immunohistochemistry to reveal the proliferation of cells. The scale bars are 20 µm. I) Quantitative analysis of BrdU areas via the digital tissue section scanner and image analysis system, *n* = 6. Data represent the mean ± SD. GSEA analysis for contrasting the gene sets associated with J) glycolysis and K) respiratory electron transport between PD model and D‐Au‐ZIF groups. L) PCA results of metabolites in PD and D‐Au‐ZIF groups in metabolomics analysis. M) Integrative pathway analysis for the effect of D‐Au‐ZIF on the brain of PD models. N) KEGG enrichment analysis of the identified differentially metabolites. The rectangular box circled the targets of interest associated with mitochondrial energy metabolism. O) Heatmap of differentially metabolites with respect to glycolysis and TCA cycle in the brain of PD and D‐Au‐ZIF groups. P) Biological electron microscopy (scale bars: 50 nm) and Q) mitochondrial staining (scale bars: 20 µm) analyses for the microglia isolated from midbrain tissue in PD models after D‐Au‐ZIF treatment.

The above studies have demonstrated the therapeutic effect of chiral NPs on the mitigation of pathological symptoms and neuroinflammation in PD models, but the specific intracellular biological effects and mechanisms were still unknown. Therefore, transcriptomic and metabolomic analyses were subsequently performed to investigate the biological effects of D‐Au‐ZIF‐mediated neuroinflammatory remission in PD models. For transcriptomic analysis, principal component analysis (PCA) results indicated a completely distinct distribution between PD model and D‐Au‐ZIF groups (**Figure** **7**A). Additionally, volcano plots indicated that among the dramatically differentially expressed genes (DEGs), 500 genes were up‐regulated and 603 genes were down‐regulated after D‐Au‐ZIF treatment (Figure 7B).

Specifically, heat map in Figure 7C showed that the DEGs associated with tricarboxylic acid (TCA) cycle/oxidative phosphorylation (OXPHOS) (Fh1, Me1, Glo1, etc.), and oxidative stress resistance (Sod1, Gpx1, Gpx3, etc.) were up‐regulated, while the DEGs related to glycolysis (Pgam2, Eno3, Nupl2, etc.) and inflammation (Plscr1, Mylk3, Nlrp3, etc.) were down‐regulated after D‐Au‐ZIF treatment. Further, Kyoto Encyclopedia of Genes and Genomes (KEGG) enrichment analysis showed that pathways enriched from DEGs were clustered in Parkinson's disease, NF‐kappa B signaling pathway, TNF signaling pathway, and cytokine−cytokine receptor interaction, most of which are neuroinflammation‐related pathways (Figure 7D). Additionally, Gene Ontology (GO) enrichment indicated that signaling pathways related to neurogenesis, neuron differentiation, cell chemotaxis, and regulation of chemotaxis were considerably altered after treatment (Figure 7E). Therefore, in view of the DEGs and signaling pathways associated with neurogenesis, we further verified and analyzed it via biochemical techniques. First, a TH and NeuN co‐immunofluorescence assay in the SNpc indicated increased TH and NeuN immunogenicity after D‐Au‐ZIF treatment compared to PD mice (Figure 7F,G). Additionally, there was also an increased PAX6 immunogenicity after D‐Au‐ZIF treatment (Figure S27, Supporting Information), which was consistent with the results in GO analysis and further confirming the effects of D‐Au‐ZIF in promoting neurogenesis. Further, BrdU, a thymidine analogue, was used to label actively dividing cells during the S phase of the cell cycle. Immunohistochemistry results demonstrated an elevation of BrdU‐positive cells after D‐Au‐ZIF treatment (Figure 7H,I).

Gene set enrichment analysis (GSEA) demonstrated that glycolysis process was prohibited, while respiratory electron transport was promoted after D‐Au‐ZIF treatment (Figure 7J,K), which was consistent with the heat map analysis in Figure 7C. Similar to transcriptomic analysis, PCA results in metabolomic analysis suggested notable distinct distribution between PD model and D‐Au‐ZIF groups (Figure 7L). Meanwhile, the up‐regulated and down‐regulated metabolites were illustrated in Figure S28 (Supporting Information). Consistent with transcriptomic analysis, integrative pathway analysis found that D‐Au‐ZIF could influence metabolic process like glycolysis and citric acid cycle (i.e., TCA cycle) (Figure 7M). KEGG enrichment analysis further screened some pathways related to mitochondrial energy metabolism, including warburg effect, citric acid cycle, glycolysis, and aspartate metabolism (Figure 7N). Specifically, metabolites associated to TCA cycle (citric acid, succinic acid, 2‐hydroxyglutaric acid, and malic acid) were increased, and metabolites related with glycolysis (lactic acid, fructose, maltose, glucose 6‐phosphate, and pyruvic acid) were decreased after D‐Au‐ZIF treatment (Figure 7O).

As an essential organelle connecting energy metabolism, oxidative stress and cellular inflammation, dysfunction of mitochondria can lead to a series of consequences, including neuroinflammation and aggravation of PD. To investigate the efficacy of D‐Au‐ZIF on mitochondria dysfunctions elicited by MPTP stimulation, the microglia of midbrain tissue were isolated after treatment and examined by biological electron microscopy and mitochondrial staining. As shown in Figure 7P, in the microglia of PD model, the mitochondrial structure is blurred, accompanied by the absence of mitochondrial crista. By contrast, mitochondrial structures recovered to normal under D‐Au‐ZIF treatment. Meanwhile, mito‐tracker staining indicated that mitochondrial morphology in the microglia of PD models was discontinuous with obvious fragmentation, while treatment with D‐Au‐ZIF dramatically improved the mitochondria impairment (Figure 7Q). Additionally, the activities of mitochondrial respiratory chain complex I and complex II were detected, and the results showed that D‐Au‐ZIF significantly elevated the activities of both respiratory chain complexes (Figure S29, Supporting Information), further explained the mechanism of mitochondrial function in promoting TCA cycle. In general, the results indicated that D‐Au‐ZIF treatment elecits reprogramming of mitochondrial energy metabolism by regulating mitochondrial dysfunction mediated by oxidative damage in PD models, systemically rectifying the imbalance in cellular energy metabolism (glycolysis, TCA cycle, and OXPHOS) in the SNpc and ST regions in PD brain. However, the biological mechanisms of chiral NPs in mediating mitochondrial energy reprogramming needs to be further elucidated by molecular biological avenues.

### 2.7 Molecular Mechanisms of Microglial Energy Reprogramming and Anti‐Inflammatory Effect Mediated by Nerve Cell Communications

**Figure 8:**
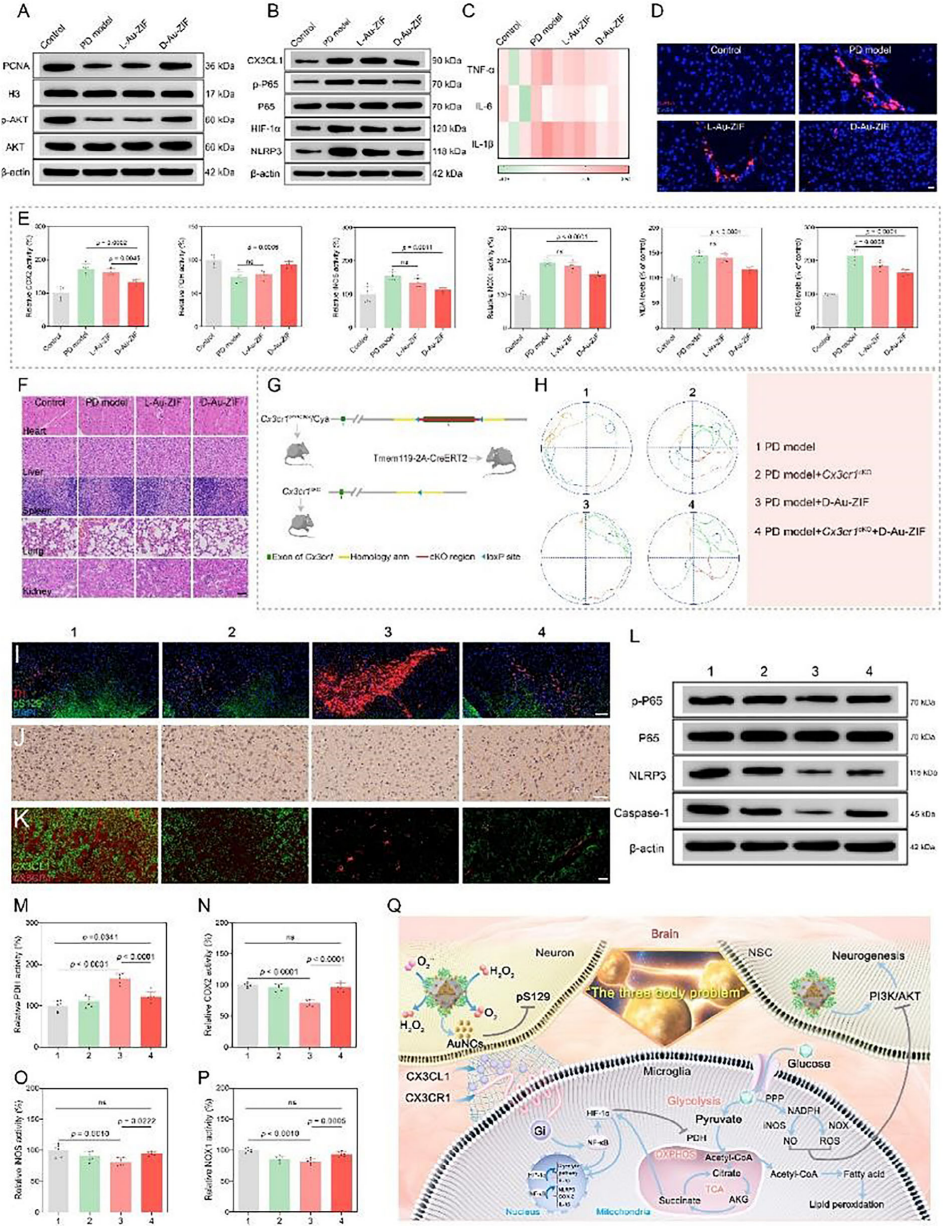
Molecular mechanism analyses of energy reprogramming in microglia mediated by chiral NPs‐induced communications among nerve cells. Western blotting showing the expression levels of proteins related to A) neurogenesis and B) the chemokine axis‐mediated NF‐κB‐NLPR3 pathway in vivo. C) Detection of the inflammatory factor expressions in the brain tissue after different treatments using qPCR. D) Fluorescence co‐staining experiments of p‐P65 and DAPI for revealing the activation of NF‐κB pathway. The scale bar is 20 µm. E) Investigation of levels of some key enzymes and factors related to oxidative damage and mitochondrial energy metabolism processes, *n* = 6. Data represent the mean ± SD. F) Hematoxylin and eosin staining of the heart, liver, spleen, lung, and kidney in the mice treated with MPTP or chiral NPs. The scale bar is 50 µm. G) Schematic diagram illustrating the breeding process of *Cx3cr1* ^cKO^ mice using the Cre‐Loxp recombinant enzyme system, in which the C57BL/6‐*Cx3cr1^em1Cflox^*/Cya mice and microglia‐specific Cre mice (Tmem119‐2A‐CreERT2) were breeded. H) The representative moving paths of *Cx3cr1* ^cKO^ PD mice after treatments. I) Co‐immunofluorescence assay of TH and pS129 in the SNpc of mice brain revealing the pathological changes in *Cx3cr1* ^cKO^ PD mice. The scale bar is 100 µm. J) Immunohistochemical assay of CD206 in the brain tissue revealing the activation of microglia. The scale bar is 50 µm. K) Co‐immunofluorescence assay of CX3CL1 and CX3CR1 in mice brain after different treatment strategies. The scale bar is 20 µm. L) Western blotting showing the expression levels of proteins related to the NF‐κB‐NLPR3 pathway in *Cx3cr1* ^cKO^ mice. M) PDH, N) COX2, O) iNOS, and P) NOX1 activities in mice brain after different treatment strategies, *n* = 6. Data represent the mean ± SD. Q) Schematic diagram illustrating that D‐Au‐ZIF interferes with the communications between neurons, NSCs and microglia, that is, D‐Au‐ZIF's antioxidant and α‐syn inhibitory activity alleviate neuronal damage, promotes neurogenesis of NSCs, and affects the energy metabolic reprogramming of mitochondria in microglia through CX3CL1/CX3CR1‐NF‐κB‐NLPR3 pathway‐mediated intercellular communications.

Given the pathways in neurogenesis and neuron differentiation identified by transcriptomic results (Figure 7E), we subsequently investigated the specific pathways of D‐Au‐ZIF in promoting NSCs differentiation. As shown in **Figure** **8**A and Figure S30A,B (Supporting Information), the expression levels of proliferating cell nuclear antigen (PCNA) and its upstream activation pathway (Akt signaling) were up‐regulated by D‐Au‐ZIF treatment, indicating the role of D‐Au‐ZIF in enhancing neurogenesis and neuron differentiation through the classic Akt signaling pathway. As shown in Figure 7D, transcriptomic results revealed that the treatment of D‐Au‐ZIF is closely related to the NF‐κB pathway. At the same time, it has been documented that NF‐κB signaling pathway is associated with the assembled of inflammasome and can interfere with intracellular glycolysis and TCA cycle through hypoxia inducible factor‐1α (HIF‐1α) and pyruvate dehydrogenase (PDH).^[^ ^34^ ^]^ Therefore, the expression of these key factors after treatment were determined. The results indicated that CX3CL1, phosphorylated P65 (p‐P65), HIF‐1α and NLRP3 were significantly elevated in PD models, which were reversed by D‐Au‐ZIF treatment (Figure 8B,D; Figure S30C‐F, Supporting Information). Meanwhile, as downstream proinflammatory cytokines, the levels of TNF‐α, IL‐6, IL‐1β (Figure 8C), and cyclooxygenase 2 (COX‐2) were down‐regulated in D‐Au‐ZIF‐treated mice, compared with elevated PDH expression (Figure 8E), which are beneficial for TCA cycle, after treatment. Next, we examined several vital markers of oxidative stress that influence glucose metabolism and TCA cycle. As shown in Figure 8E, in PD model, the expression of inducible nitric oxide synthase (iNOS), nicotinamide adenine dinucleotide phosphate oxidase 1 (NOX1), malondialdehyde (MDA), and ROS showed upward trends, which were obviously inhibited by D‐Au‐ZIF. Additionally, no significant pathological features could be observed in the paraffin sections of major organs including heart, liver, spleen, lung, and kidney (Figure 8F). Meanwhile, compared to the control group, the expressions of alanine transaminase ALT (Figure S31A, Supporting Information), aspartate aminotransferase AST (Figure S31B, Supporting Information), blood urea nitrogen BUN (Figure S31C, Supporting Information), and creatinine CRE (Figure S31D, Supporting Information) in the serum of mice was not notably altered after the administration of the chiral NPs, indicating their satisfactory biocompatibility and biosafety for future clinical applications.

Given the cytokine‐cytokine receptor interaction pathway identified by KEGG enrichment analysis (Figure 7D), as well as the cell chemotaxis and regulation of chemotaxis pathways identified by GO enrichment analysis (Figure 7E), we continued to explore the role of the chemokine axis, which is associated with cell communications between neurons and microglia and neuroinflammation events, in the treatment of D‐Au‐ZIF. The franctalkine (CX3CL1)/CX3CR1 axis is the most relevant involving neurons and microglia. The ligand CX3CL1 is expressed and secreted by neurons while its receptor CX3CR1 is in microglia.^[^ ^35^ ^]^ Therefore, conditional knockout (cKO) of *Cx3cr1* gene in the brain of mouse, named *Cx3cr1* ^cKO^ mouse, was constructed for the subsequent studies (Figure 8G). First, the Morris water maze test showed an improved target quadrant occupation in PD mice after D‐Au‐ZIF treatment, but this improvenment was abrogated in *Cx3cr1* ^cKO^ PD mice (Figure 8H; Figure S32A, Supporting Information). A similar situation also occurred in the rotatory‐rod and pole‐climbing tests, that is, the knockout of *Cx3cr1* inhibited the remission effect of D‐Au‐ZIF on PD behavioral disorders (Figure S32B–E, Supporting Information). Additionally, pathological evaluation revealed that the level of TH was increased, while the levels of pS129 (Figure 8I; Figure S32F,G, Supporting Information) and CD206 (a proinflammatory marker of microglia) (Figure 8J; Figure S32H, Supporting Information) were decreased in D‐Au‐ZIF‐treated PD mice. However, these improvements were counteracted to levels close to those in the PD models in *Cx3cr1* ^cKO^ PD mice. Further, we performed immunofluorescence assay to detect the state of CX3CL1 and CX3CR1 under different treatment modes. Results shown in Figure 8K indicated that D‐Au‐ZIF dramatically decreased the expression and secretion of CX3CL1, but there was a partial recovery of CX3CL1 expression in *Cx3cr1* ^cKO^ mice.

Similarly, we also examined the protein expressions associated with the NF‐κB‐NLRP3 pathway. The results demonstrated that *Cx3cr1* knockout significantly affected the inhibitory effect of D‐Au‐ZIF on NF‐κB‐NLRP3 pathway, and the corresponding inflammasome assembly and expressions of inflammatory factors were up‐regulated (Figure 8L). Further, we also examined the effect of chemokine axis on reprogramming of mitochondrial energy metabolism. As shown in Figure 8M–P and Figure S33 (Supporting Information), Knockout of *Cx3cr1* significantly affected the effect of D‐Au‐ZIF on mitochondrial energy reprogramming, which was manifested by the changes in activity/level of several key factors in mitochondrial metabolism. Collectively, combining the metabolomics, transcriptomics, and biochemical analyses in Figures 7 and 8, we can cautiously draw the following conclusions: in the therapeutic process of PD, the effect of D‐Au‐ZIF on neuroinflammation relief results from the reprogramming on the energy metabolism of microglia mitochondria, which is closely related to communications between nerve cells. Through our research, we found that the communications between nerve cells including but not limited to the following aspects: D‐Au‐ZIF inhibits the pathological protein pS129 while efficiently alleviating oxidative damage in neurons, thereby affecting the secretion of chemokine CX3CL1 and interfering with the NF‐KB pathway in microglia mediated by the chemokine axis CX3CL1‐CX3CR1; This leads to a series of cascade reactions in microglia, resulting in reprogramming of mitochondrial energy metabolism; Meanwhile, the release of NO, ROS and inflammatory factors by microglia also interferes with the PI3K/AKT signaling pathway in NSCs, thus interfering with neurogenesis. Therefore, the complex communications between neurons, NSCs and microglia form an interaction similar to “the three‐body problem”, which is jointly responsible for the molecular mechanisms of D‐Au‐ZIF in mitigating neuroinflammation in the treatment of PD (Figure 8Q).

## 3 Conclusion

In summary, based on the lack of chirality and the deficiency in anti‐inflammatory capacity per unit mass of ZIF‐based NPs found in our previous studies, we designed ZIF assembly of chiral AuNCs and explored the mechanism of chirality‐mediated metabolism and BBB traversing in vivo. Proteomic and biochemical analyses indicated that chiral NPs adsorb different protein components (lipoproteins, immunoglobulins, and complement proteins) in the blood, resulting in a different protein corona composition on their surface, which determines their circulation time in vivo and the efficiency of traversing the BBB. The results of in vivo therapeutic effect are consistent with the above studies, that is, D‐Au‐ZIF with higher efficiency in BBB traversing has better effect in mitigating PD symptoms. Additionally, the results of multi‐omics analyses in the therapeutic effect of D‐Au‐ZIF suggested the key role of chemokines, neurogenesis, and neuroinflammation in the treatment of PD. Molecular biology techniques further revealed that the neuroinflammatory relief arises from the “the three‐body problem” among neurons, NSCs and microglia: the super antioxidant activity of D‐Au‐ZIF improves the antioxidant capacity of neurons, reduces the aggregation of pathological proteins and the secretion of CX3CL1, and communicates with microglia through the chemokine axis (CX3CL1/CX3CR1); The chemokine axis initiates a series of signaling pathways such as NF‐κB‐NLRP3 in microglia, mediates mitochondrial energy reprogramming, and further improves redox homeostasis in the brain microenvironment; Through the PI3K/AKT signaling pathway, the redox microenvironment of microglia and neurons affects the neurogenesis of NSCs and the proliferation of neurons. In conclusion, these data provide proof‐of‐concept that chiral nanomaterials are successfully developed against PD through differential metabolism, as well as the therapeutic mechnism in “the three‐body problem” regulating nerve cell communications.

## 4 Experimental Section

### Animal Ethics Statement

All animal studies were approved by the Institution Animal Ethics Committee of Zhengzhou University (license No. ZZU‐LAC20240617[9]).

### Statistical Analysis

All statistical analyses were conducted using the GraphPad Prism 8.0.2 in a blinded manner. The differences in means between different groups were compared via one‐way ANOVA (with Tukey's post hoc correction for multiple comparisons). Data represent the mean ± SD. The specific statistical analysis results for each experiment (e.g., sample size (n) for each statistical analysis, and P value) were shown in the figures, and “ns” indicates not significant.

## Conflict of Interest

The authors declare no conflict of interest.

## Supporting information



Supporting Information

## Data Availability

The data that support the findings of this study are available from the corresponding author upon reasonable request.
