# Supplementary material for: Communications Among Neurocytes in Parkinson's Disease Regulated by Differential Metabolism and Blood‐Brain Barrier Traversing of Chiral Gold Cluster‐MOF Integrated Nanoparticles
Source: Adv Sci (Weinh). 2025 May 14;12(23):2500026. doi: 10.1002/advs.202500026 (PMC12199330; doi:10.1002/advs.202500026)
Supplement: Supplementary file 1 — Supporting Information [file ADVS-12-2500026-s001.docx]

**Supplementary Information**

**Communications among neurocytes in Parkinson****'s disease regulated by differential metabolism and blood-brain barrier traversing of chiral gold cluster-MOF integrated nanoparticles**

*Junyang Chen^#^, Gaoxiang Xu^#^, Runpu Shen,* *Jianzhong Xu, Congcong Lu, Xin Li, Qi Feng, Qing Li**

Miss. C. Lu, Dr. X. Li, Dr. Q. Feng, and Prof. Q. Li

Department of Neurology, The Second Affiliated Hospital of Zhengzhou University, Zhengzhou University, Zhengzhou 450052, China.

Email: [sahlq@zzu.edu.cn](mailto:sahlq@zzu.edu.cn)

Dr. J. Chen

School of Life Sciences, Zhengzhou University, Zhengzhou 450001, China.

Dr. G. Xu, and Dr. J. Xu

Department of Orthopedics, The First Affiliated Hospital of Zhengzhou University, Zhengzhou 450052, China.

Dr. R. Shen

Zhejiang Engineering Research Center of Fat-soluble Vitamin, College of Chemistry and Chemical Engineering, Shaoxing University, Shaoxing, Zhejiang 312000, P. R. China.

Dr. Q. Feng

Department of Nephrology, the First Affiliated Hospital of Zhengzhou University, Zhengzhou 450052, China

*^#^* These authors contributed equally to this work.

*** Corresponding author.

**Experimental Section**

**Materials.** The following chemicals were acquired from Sangon Biotech Ltd (Shanghai, China): L-Glutathione (GSH), D-Glutathione, and gold chloride trihydrate. 5,5-Dimethyl-1-pyrroline N-oxide (DMPO) and 2-Methylimidazole (2-MIM, 98%) was purchased from Aladdin Reagent Co. Ltd (Shanghai, China). Xanthine oxidase was purchased from Shanghai yuanye Bio-Technology Co., Ltd. Zinc nitrate hexahydrate (Zn (NO_3_)_2_·6H_2_O) was purchased from Fuchen Chemical Reagent Co., Ltd (Tianjin, China). We used ultrapure water with resistivity higher than 18.2 MΩ cm to prepare aqueous solutions. DMEM and FBS were purchased from Gibco. SH-SY5Y cells were purchased from Pricella (CL-0208). Thiazolyl blue tetrazolium bromide (MTT) was purchased from Sigma-Aldrich. The mitochondrial membrane potential assay kit with JC-1 (C2006) and Reactive oxygen species assay kit (S0033S) were purchased from Beyotime (Shanghai, China). Mouse PDH detection kit (CB10207-Mu), mouse iNOS detection kit (CB10817-Mu), and mouse NOX1 detection kit (CB12394-Mu) were purchased from COIBO (Shanghai, China). ROS (E004) and MDA detection kit (A003) were purchased from Jiancheng Bioengineering Institute (Nanjing, China). DMEM and FBS were purchased from Gibco. SH-SY5Y cells were purchased from Pricella (CL-0208). Thiazolyl blue tetrazolium bromide (MTT) was purchased from Sigma-Aldrich. The mitochondrial membrane potential assay kit with JC-1 (C2006) and Reactive oxygen species assay kit (S0033S) were purchased from Beyotime (Shanghai, China). Mouse PDH detection kit (CB10207-Mu), mouse iNOS detection kit (CB10817-Mu), and mouse NOX1 detection kit (CB12394-Mu) were purchased from COIBO (Shanghai, China). ROS (E004) and MDA detection kit (A003) were purchased from Jiancheng Bioengineering Institute (Nanjing, China).

**Instruments.** The morphology of the nanoparticles was characterized by a transmission electron microscope (Tecnai G2 F20 S-TWIN TMP) operating at an acceleration voltage of 200 kV. The X-ray diffraction (XRD) patterns were acquired on a Bruker AXS D8 Advance Powder X-ray diffractometer (Bruker, Germany). The UV-vis absorbance was obtained by using a U-3900H spectrometer (Shimadzu, Japan). Fluorescence measurements were performed on an F-7100 spectrophotometer (Hitachi, Japan). Size distributions were determined using Malvern Zetasizer Nano ZS90. Circular dichroism (CD) measurements were conducted on a Chirascan CD Spectrophotometer. The quantification of Au was measured by inductively coupled plasma optical emission spectrometer (Shimadzu, Japan). EPR spectroscopy was carried out by an EPR spectrometer (Bruker, Germany).

**Preparation of L-/ D-/ L/D-Gold Nanoclusters.** The L-/ D-/ L/D-gold nanocluster (L-/ D-/ L/D-Au) was prepared according to our previous report.^[1,2]^ In brief, HAuCl_4_ (25 mM) was slowly added into 1 mL of L-GSH peptide (15.4 mg) solution at room temperature. After sufficient agitation, the reaction was continued in a water bath at 70 °C for 12 h and then placed in the dark for 12 h. For the synthesis of D-Au, only L-GSH was replaced by D-GSH. The resultant products were washed and condensed by ultrafiltration at 6000 rpm for 30 min (5 times). The obtained gold nanocluster were finally dispersed in an aqueous and stored in the refrigerator (4 °C) for further research. The achiral L/D-Au were prepared using D-GSH/L-GSH (molar ratio=1:1) as the reducing agents.

**Synthesis of L-/ D-/ L/D-Au@ZIF NPs.** In a typical experiment, 2-methylimidazole (328 mg) in 7.5 mL methanol were mixed with 2 mL L-Au (2 mg/mL) under constant stirring, followed by the addition of Zn(NO_3_)_2_·6H_2_O (149 mg, 5.5 mL) solution. After stirring for about 5 hours, the product of L-Au@ZIF was collected by centrifugation (7,500 rpm, 5 min). After washed with methanol for three times, the L-Au@ZIF was dried at 60 °C and stored at 4 °C for further using. The D-Au@ZIF or L/D-Au@ZIF were prepared with similar process, only replacing L-Au to D-Au or L/D-Au.

**Synthesis of L-/ D-/ L/D-Au-ZIF NPs.** Au@ZIF NPs (20 mg) was dissolved in 5 mL water. Then, 5 mL Au nanoclusters (0.5 mg/mL) was dropwise added into Au@ZIF NPs solution under sonication condition. After ultrasonicated at room temperature about 30 mins, the product was collected by centrifugation (7,500 rpm, 5 min). The collected production was dried at 60 °C and stored at 4 °C for further using. The D-Au-ZIF or L/D- Au-ZIF were prepared with similar process, only replacing L-Au@ZIF and L-Au to D-Au@ZIF and D-Au or L/D-Au@ZIF and L/D-Au.

**Total antioxidant capacity test (ABTS rapid method).** The total antioxidant capacity of clusters was determined by the rapid ABTS method using a prepared ABTS^•+^ aqueous solution. The antioxidant capacity was analyzed by monitoring the absorption value at 734 nm using a UV-vis spectrophotometer. For the determination of antioxidant activity, 1 mL of ABTS reagent was mixed with 100 μL of either nanozyme samples (100 μg/mL) or PBS buffer. The absorbance was then continuously measured at 734 nm for 10 mins.

**SOD-like Test.** The •O_2_^−^ elimination rates were used to calculate their SOD-like activity. Nitroblue tetrazolium (NBT) was used as an indicator for •O_2_^−^, which was produced by the xanthine-xanthine oxidase (X-XOD) system. The control system consisted of 0.25 mL xanthine, 0.25 mL NBT, 0.1 mL xanthine oxidase, and 0.5 mL PBS buffer. The •O_2_^−^ concentration of the control system was determined by measuring the increase in absorbance at 560 nm (ΔA1) of NBT using an ultraviolet spectrophotometer for 10 minutes. Nanoparticles with different concentrations were individually added to the control system, and the increase in absorbance at 560 nm (ΔA2) of NBT was recorded for 10 minutes. The •O_2_^−^ elimination rate was calculated using the formula: (ΔA1 - ΔA2) / ΔA1 * 100%.

**CAT-like test.** The CAT-like activity of NPs was tested by monitoring the characteristic absorption value of H_2_O_2_ at 240 nm using a UV-vis spectrophotometer. The optical density decreases with the decomposition of H_2_O_2_ resulting from the CAT-like activity of NPs. The reaction solutions contained 50 μg/mL nanozyme and 0.3% H_2_O_2_ in PBS buffer solution.

**Scavenge •OH and •O_2_^−^ by EPR spectra analysis.** First, •OH was generated through Fenton reaction with Fe^2+^/H_2_O_2_ system by 5 mM FeSO_4_ and 10 mM H_2_O_2_ for 10 min. In addition, •O_2_^−^ was generated from the production of xanthine (5 mM) and xanthine oxidase (1 U/mL) at room temperature for 30 min. Then, the •OH and •O_2_^−^ scavenging abilities of L-Au-ZIF, D-Au-ZIF, and L/D-Au-ZIF were examined by EPR spectra analysis: L-Au-ZIF, D-Au-ZIF, and L/D-Au-ZIF (50 μg/mL) were added into the obtained •OH or •O_2_^−^ radical solution and incubated for another 30 min. After that, DMPO was added to the mixture solution for 10 min using EPR to detect the ability of three NPs to scavenge •OH and •O_2_^−^ radical.

**Computational details.** All geometric structures were optimized using the ORCA 6.0.0 program package. The BP86 functional in combination with Grimme’s dispersion correction (D3) and Becke−Johnson damping factor (BJ) were used during geometry optimization, vibrational analysis, while the ω97M-V hybrid functional was used during single point calculations. The def2-svp basis set was used for non-metal elements while SARC-ZORA-SVP for Au were used during optmization and vibrational frequency analysis, while def2-tzvp basis set was used for non-metal elements while SARC-DKH-TZVPP for Au during single point calculations. To reflect the influence of water as a solvent, the CPCM and SMD implict solvation model were used during geometry optimization and single point calculations respectively. All analysis were carried out using the Multiwfn 3.8 Dev software package and visualized by VMD 1.9.3.

***In vitro* cytotoxicity and** **neuroprotective effect analysis.** The *in vitro* cytotoxicity of chiral NPs and neuroprotective effect were evaluated by MTT method on SH-SY5Y and BV2 cells. Cells (1×10^4^ cells per well) were seeded into 96-well plates. Various concentrations of chiral NPs were added into wells for 6 h, after which 2 mM MPP^+^ or 100 ng/mL LPS were added and incubated for 24 h. Subsequently, 20 μL of MTT solution (5 mg/mL) was added into each well and incubated at 37 °C for additional 4 h. Finally, the formanzan crystal was dissolved in dimethyl sulfoxide (DMSO) and the absorbance at 492 nm was detected using a GF-M3000 microplate reader (CAIHONG, China).

***In vitro* ROS levels.** ROS contents in SH-SY5Y cells were analysed using DCFH-DA fluorescent dye. Briefly, cells were seeded into 6-well plates with densities of 1.5 × 10^5^ cells per well and chiral NPs (80 μg/mL) were added into the plates for co-culturing for 6 h. Then, cells were exposed to 2 mM MPP^+^ for 2 h in the incubator, after which DCFH-DA (8 μM) was added and stained for 30 min. ROS expression was intuitively observed by Olympus FV1200 laser scanning confocal microscope (Tokyo, Japan).

**Quantitative real-time PCR (qPCR).** The brain tissues were used for RNAs extraction with Trizol Reagent, after which the cDNA was generated by PrimeScript^®^ RT reagent Kit (RR047A, TaKaRa, Japan). Then, the expression levels of *TNF-α*, *IL-1β*, *IL-6,* and *IL-10* were detected on 7500 Real-Time PCR System (Applied Biosystems, Canada) with TB Green Premix Ex Taq^TM^ Ⅱ kit (RR820A, Takara, Japan), after which the data were calculated by the 2^−ΔΔCT^ method using *β-actin* as a control gene for normalization.

**Mitochondrial membrane potential.** JC-1 dye was used for detecting mitochondrial membrane potential. Briefly, SH-SY5Y cells were seeded into plates with densities of 2.0 × 10^5^ cells per well and cultured overnight. Subsequently, cells were incubated with chiral NPs (80 μg/mL) for 6 h and then treated with 2 mM MPP^+^ for 24 h, followed by the addition of JC-1 probe at room temperature for 40 min. Finally, the mitochondrial membrane potentials were analyzed by Olympus FV1200 laser scanning confocal microscope.

**Parkinson’s Disease (PD) mouse model and animal studies.** The male C57BL/6J mice (21~25 g, 8-week-old) were purchased from the Vital River Laboratory Animal Technology Co., Ltd. (Beijing, China). All mice were fed in cages with controlled temperature and humidity, and kept free access to water and food. Before subsequent experiments started, all mice were left to adapt for 5 days in the animal house with 12 h light/dark cycles to simulate the natural environment of day and night. Mice were subjected to intraperitoneal MPTP administration (35 mg/kg/day for consecutive 7 days) for inducing a PD-like phenotype, and were randomly divided into 4 groups: healthy mice (control), PD model, PD model+L-Au-ZIF (L-Au-ZIF), and PD model+D-Au-ZIF (D-Au-ZIF). L-Au-ZIF and D-Au-ZIF (2 mg/kg/day) were intravenously injected into mice every other day for 5 times after MPTP injection. PD model and control groups received saline only. Mice were subjected to behavioral testing on the fifth day after the last injection.

**Blood clearance rate.** PD mouse models were divided into three groups (n = 3), and were respectively injected with L/D-Au-ZIF, L-Au-ZIF, and D-Au-ZIF (2 mg/kg). Intravenous blood was taken at various times after injection. The blood container was an anticoagulant tube with heparin sodium added. Immediately after blood collection, the samples were centrifuged (600 g) at 4 ℃ for 10 min to obtain plasma and transferred to a new EP tube. Finally, the contents of NPs in plasma were measured by the SOD-like activity and the half-lives of enzymatic activities was calculated.

**Preparation of NPs-protein corona samples.** The process of obtaining mice plasma samples is the same as that of “blood clearance rate”, after which the plasma samples were diluted to 20% with phosphate buffer. Then, 150 μL NPs (5.0 mg/mL) and 1 mL 20% mice plasma were mixed in a 2.5 mL centrifuge tube and left for different time at room temperature. Subsequently, the resulting mixture is centrifuged (8000 g, 30 min) at 4 ℃ to remove the supernatant and the precipitate was collected. Then, 1 mL of PBS buffer was added, and the particles were re-suspended by ultrasonic methods. The above steps were repeated for five times until a sample of NPs-protein corona with high affinity to NPs was obtained. Finally, the obtained samples were re-dispersed in phosphate buffer and stored at -80 ℃ for use.

**Proteomics analysis.** The samples were concentrated with ultrafiltration tubes and added 50 μL cracking solution composed of SDS, DTT, and Tris HCl to disperse the concentrated liquid and transfer it to the centrifuge tube. After Boiling with water bath for 5 min, the samples were centrifuged for supernatant at 4 ℃. Appropriate amount of peptide segment was selected from the enzymolysis sample and then used in nanofervation chromatographic separation. Afterwards, the sample was injected into trap column, after which gradient separation was carried out by chromatographic analysis column. For mass spectrometry analysis, each scan cycle contains one MS full scan, and the following 40 MS/MS scans. The mass spectrum data were searched by the Protein Discover software, and the database was the proteome reference database of mice in Uniprot.

**Distributions in the brain and major organs.** For the inductively coupled plasma mass spectrometry (ICP-MS) analysis, PD models were injected with L/D-Au-ZIF, L-Au-ZIF, and D-Au-ZIF (2 mg/kg) through the tail vein (n = 6 for heart, liver, spleen, lung, kidney, and n = 12 for the brain). 24 h after administrations, the mice in each group were sacrificed, and major organs and the brain were collected. The tissues were weighed and homogenized to get the percentage of injected dose per gram of tissue (% ID g^-1^) by ICP-MS (Agilent 7800, China). For the analysis of tissue penetration ability, The chiral NPs with the same dose (2 mg/kg/time) were injected intravenously into the mice every two hours for a total of three times before the brain tissues were taken. The brains of PD models were collected and fixed with 4% paraformaldehyde. Afterwards, the tissues were embedded in paraffin for sections with 10 μm thickness. Afterwards, the tissue sections were observed and photographed by panoramic section scanner. The 3D mode and line scan mode of Image J were used for 3D analysis of tissue sections and the fluorescence intensity analysis along the axis from cerebral cortex to cerebral parenchyma.

**Lysosome co-localization.** Briefly, bEnd.3 cells were cultured in 6-well plates with a density of 1.5×10^5^ cells/well and cultured in a cell incubator overnight. The cells were stained by Hoechst 33342 (blue) and Lyso Tracker (red), and then incubated with FBS-free DMEM containing L-Au-ZIF, and D-Au-ZIF (80 μg/mL) for 2, 4, 6, and 8 h, respectively. Finally, the samples were used for the detection on a laser scanning confocal microscope to analyze the co-localization of chiral NPs with lysosomes.

**Intracellular internalization mechanisms.** Briefly, bEnd.3 cells were inoculated in 96-well plates at an initial density of 8×10^3^ cells/well and cultured at 37 °C. Then, the cells were cultured with chiral NPs (80 μg/mL) for 4 h in different endocytosis inhibitor conditions. Afterwards, the cells were washed with PBS five times and subjected to fluorescence intensity detection *via* a microplate reader.

**Behavioral evaluations.** A vertical pole (d: 1 cm, h: 50 cm) was used for the pole-climbing, and time taken by the mice to turn head downward (T-turn), as well as the time mice taken to reach the bottom of the pole (T-total) were recorded. For the rotatory-rod test, mice were placed on the rotating rod for 2 min. Two vital indicators in the test including latency to fall and total drops were recorded. Both the pole-climbing test and rotatory-rod test were performed thrice to ensure accuracy.

**Histological analysis.** After the mice were sacrificed, the mice brains in each group (n = 6) were collected and fixed with 4% paraformaldehyde (PFA) for 72 h. After subjecting to embedding in paraffin and sectioning, antigen retrieval was realized by EDTA (PH = 9.0) with medium fire for 8 min to boiling, ceasing fire for 8 min and then turning to medium-low heat for 7 minutes. Afterwards, the sections were subjected to 3% H_2_O_2_ for 25 min to block endogenous peroxidase activity. Blocking was accomplished with 3% BSA for 30 min at room temperature. The sections were incubated with primary antibodies against TH (GB12181, Servicebio, dilution: 1:1000), pS129 (ab51253, Abcam, dilution: 1:500), GFAP (GB12096, Servicebio, dilution: 1:1000), Iba-1 (GB12105-100, Servicebio, dilution: 1:1000), PAX6 (ab195045, Abcam, dilution: 1:500), BrdU (ab6326, Abcam, dilution: 1:200) at 4 ℃ overnight, and subsequent fluorescent- or HRP-labeled secondary antibody for 50 min at room temperature. After dehydration and sealing processes, the sections were photographed by panoramic section scanner (Pannoramic, 3DHistech).

**Transcriptomics analysis.** Trizol Reagent (Invitrogen, USA) was used to obtain the total RNA in brain tissues, and Oligo (dT) magnetic beads were used to enrich mRNA with polyA structure among total RNA. All RNA was interrupted to fragments in 200 ~ 300 bp by ion-interruption. After the library was constructed, PCR amplification was applied to enrich the library fragments, and then the library was selected according to the fragment size. Afterwards, the total concentration and effective concentration of the library were detected through 2100 Bioanalyzer (Agilent, USA). After RNA extraction, purification, and library construction, the library was subjected to paired-end sequencing using the Next-Generation Sequencing (NGS) based on Illumina HiSeq X10 (Illumina, San Diego, USA). TopGO (<http://www.bioconductor.org/packages>) and KAAS (<https://www.genome.jp/tools/kaas/>) were used for bioinformatics analyses.

**Metabolomic analysis.** The metabolomic analysis was performed based on high resolution liquid chromatography coupled to mass spectrometry (LC-MS) using chromatograph (UltiMate 3000 RS, Thermofisher, China) and mass spectrometer (Q Exactive, Thermofisher, China). All the raw data were processed, peak integrated, comparison, and quantified by TMBQ software (v1.0, Metabo-Profile, China). Further individual metabolites, pathway and enrichment analysis were performed with MetaboAnalyst 4.0 (http://[www.metaboanalyst.ca](http://www.metaboanalyst.ca)).

**Western blotting.** The concentrations of protein samples were detected using BCA assay (G2026, Servicebio). Afterwards, the samples were subjected to separation and transfer using SDS-polyacrylamide gels and PVDF membrane before blocking with 5% BSA in PBST. Then, the samples were incubated with primary antibody including anti-pS129 (ab51253, Abcam, dilution 1:2000), anti-PCNA (GB11010, Servicebio, dilution 1:1000), anti-Histone H3 (GB11102, Servicebio, dilution 1:1000), anti-Akt (GB15689, Servicebio, dilution 1:1000), anti-p_(Ser473)_ Akt (12694, CST, dilution 1:1000), anti-CX3CL1 (ab25088, Abcam, 2 μg/mL), anti-P65 (ab32536, Abcam, dilution 1:2000), anti-p-P65 (ab76302, Abcam, dilution 1:1000), anti-HIF-1α (ab1, Abcam, 5 μg/mL), anti-NLRP3 (ab263899, Abcam, dilution 1:1000), anti-Caspase-1 (GB11383, Servicebio, dilution 1:1000) for 24 h, and then secondary antibody labelled with HRP (GB23303/GB23301, Servicebio). Subsequently, the target antigens were imaged by chemiluminescence imaging system (SCG-W2000, Servicebio).

**Isolation of microglia.** The mice midbrain tissue was separated and homogenized by adding 5 mL PBS in a homogenizer, and then the homogenized tissues were filtered by a 70 μm cell filter. The obtained cells masses were centrifuged (1000 g, 10 min) at 4 ℃ and then re-suspended with 5 mL 70% Percoll. After centrifugation at 800 g for 30 min, the myelin-rich upper layer was removed, the middle layer was obtained and mixed with 40 mL pre-cooled PBS, and then centrifugation (1400 g, 5 min) at 4 ℃ to get microglia-rich cell masses. Afterwards, the isolated microglia were subjected to biological electron microscope analysis and mito-tracker fluorescence staining analysis.

***Gene* knockout of *Cx3cr1*.** The *Cre-Loxp* recombinant enzyme system was applied for the specific knockout of *Cx3cr1* gene in the brain of C57BL/6 mice. In C57BL/6-*Cx3cr1^em1Cflox^*/Cya mice (S-CKO-01949, Cyagen, China), the upper and lower reaches of the second exon of *Cx3cr1* were inserted into two Loxp locus in the same direction, and deletion of this region should result in the loss of function of the mouse *Cx3cr1* gene. The purchased C57BL/6-*Cx3cr1^em1Cflox^*/Cya mice mice were breeded with microglia-specific Cre mice (Tmem119-2A-CreERT2, Cyagen, China) under tamoxifen treatment. The mice whose progeny were flox homozygous and carried Cre were identified as the controlled knockout mice that specifically knocked out microglia *Cx3cr1*, namely *Cx3cr1*^cKO^ mice.

**Biosafety evaluation.** Chiral NPs (2 mg/kg/day) or saline (control group) were intravenously injected into C57BL/6 male mice every other day for 5 times. The mice in MPTP group were subjected to intraperitoneal injection of MPTP (35 mg/kg/day) for consecutive 7 days. Afterwards, all mice were sacrificed and organs including heart, liver, spleen, lung, and kidney were obtained. After fixing in 4 % paraformaldehyde for 48 h, the tissues were subjected to gradient dehydration with ethanol solutions. Then, the tissue blocks were embedded with melted paraffin wax after treating twice in xylene**.** The wax blocks were sliced *via* a microtome (RM2016, Leica, China), and the slices were stained with hematoxylin and eosin (H&E) and photographed by fluorescence microscope. Additionally, the blood samples of mice were collected and allowed to stand at room temperature for 30 min, after which the samples were centrifuged at 800 *g* for 15 min. Afterwards, the supernatant serum samples were obtained for hematological biochemical analyses including alanine transaminase (ALT), aspartate aminotransferase (AST), blood urea nitrogen (BUN) and creatinine (CRE).

**Supplementary Figures**


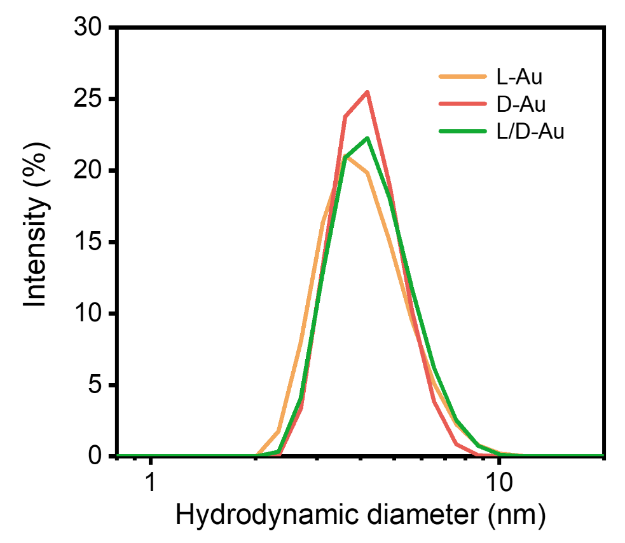


**Figure S1.** The hydrodynamic diameters of L-AuNCs, D-AuNCs and L/D-AuNCs.


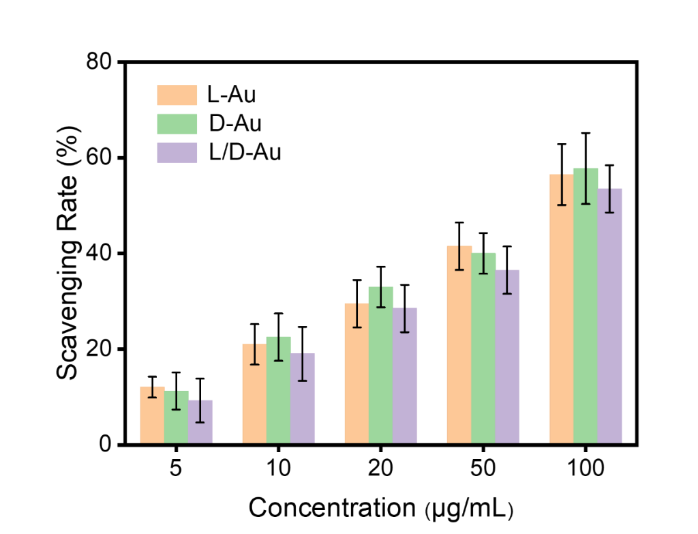


**Figure S2.** The SOD-like activities of L-Au


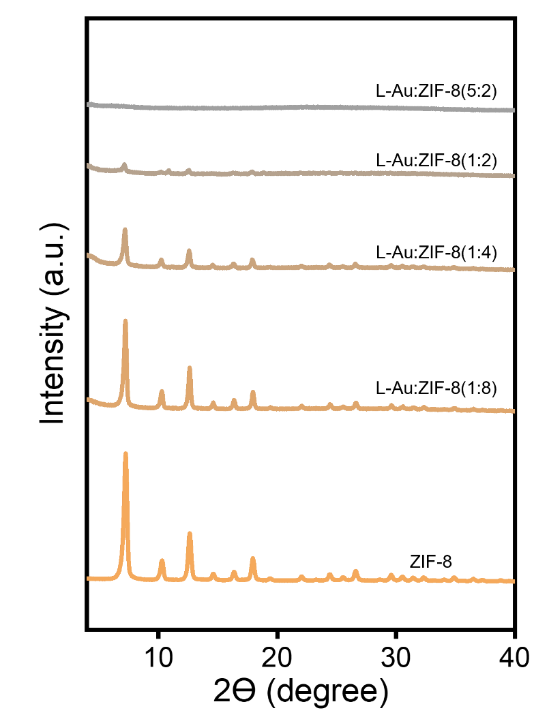


**Figure S3.** XRD curves of L-Au&ZIF nanocomposites formed with the L-Au/ZIF-8 mass ratio of 0:1, 1:8, 1:4, 1:2, and 5:2.


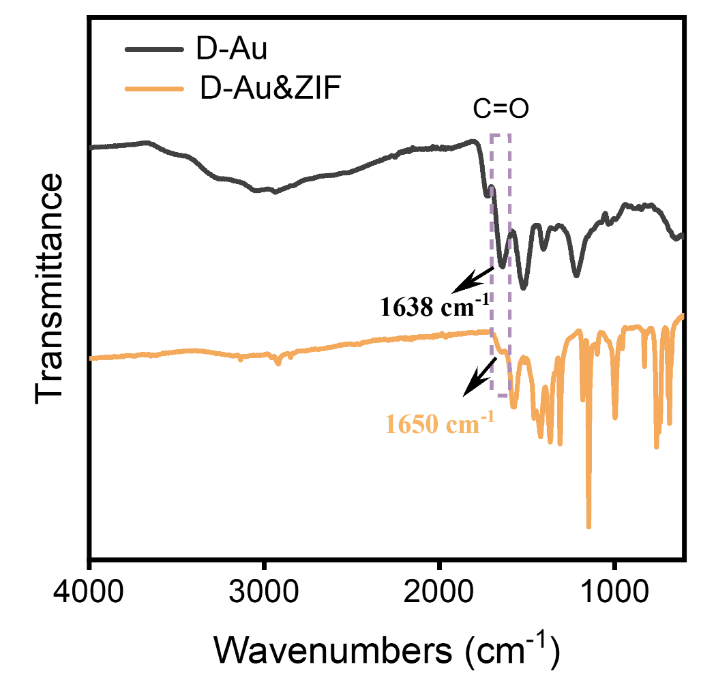


**Figure S4.** The FTIR of D-Au and D-Au&ZIF.


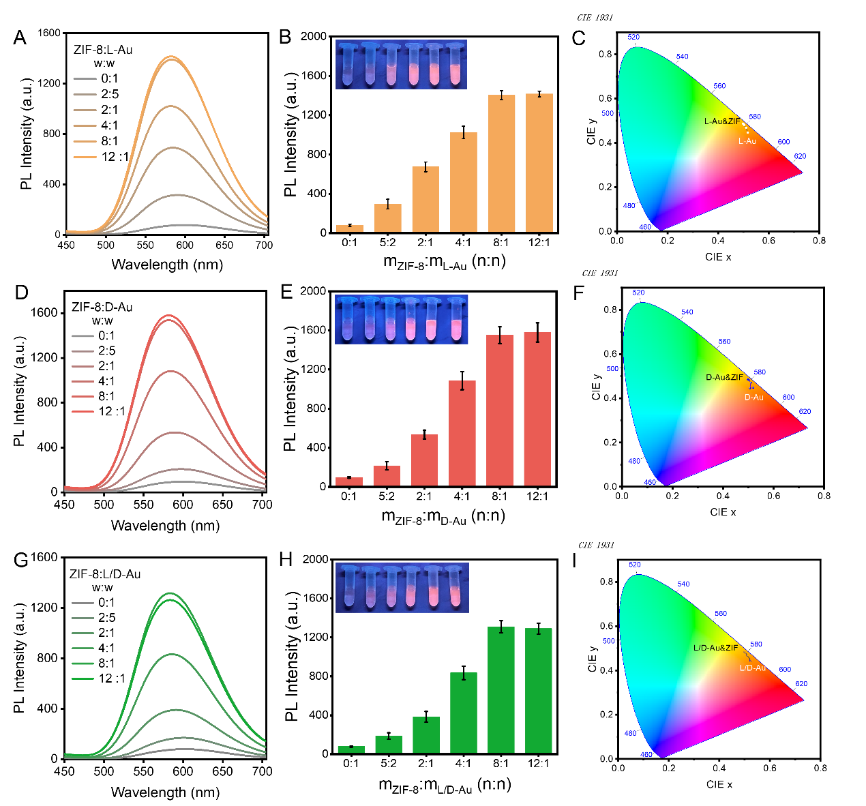


**Figure S5.** Fluorescence emission spectra, corresponding intensity and digital photos, and CIE chromaticity charts (A, B, C) L-Au&ZIF, (D, E, F) D-Au&ZIF, and (G, H, I) L/D-Au&ZIF at various weight ratios of ZIF-8 to AuNCs.


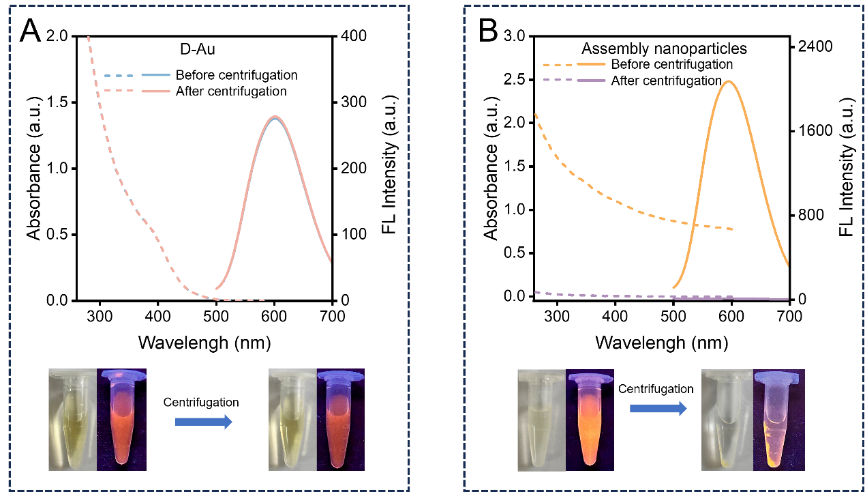


**Figure S6.** The UV-vis and Fluorescence spectra of (A) AuNCs and assembled D-Au-ZIF nanoparticles before and after centrifugation (8,000 rpm for 10 min), bottom: corresponding picture taken under daylight and an UV lamp, respectively.


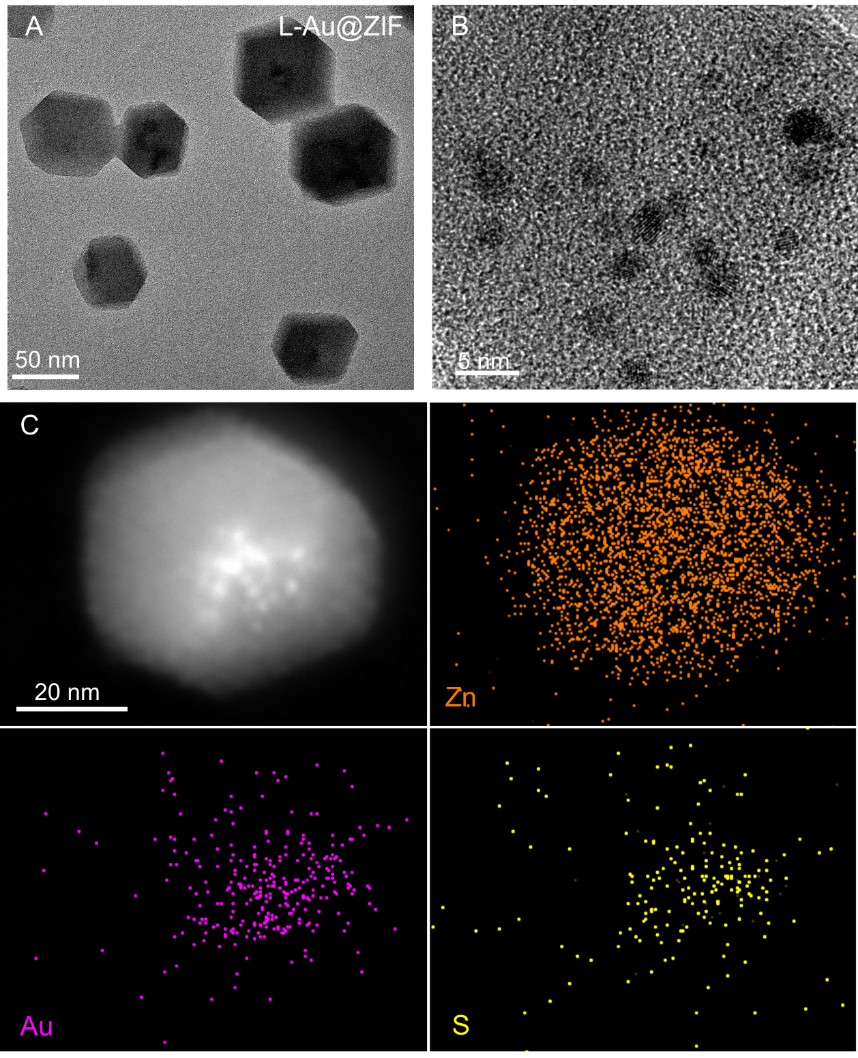


**Figure S7.** (A) TEM, (B) HRTEM, and (C) EDS mappings images of L-Au@ZIF.


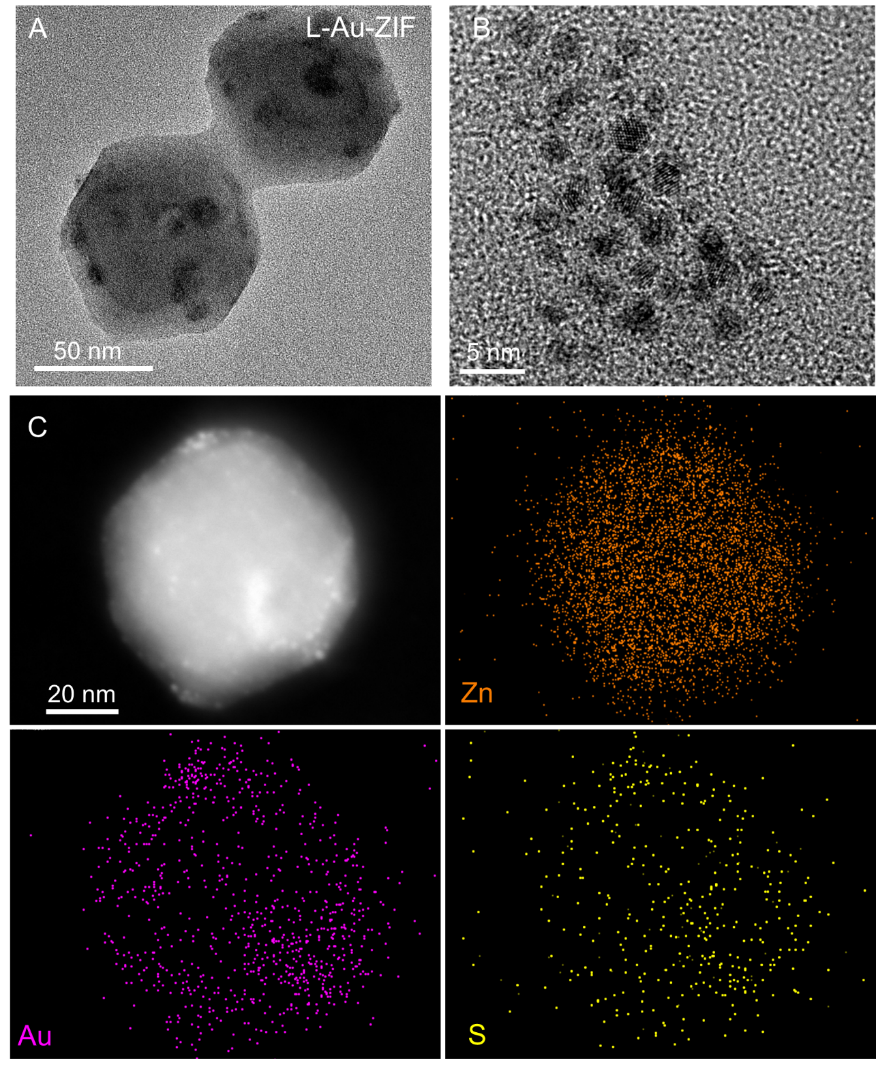


**Figure S8.** (A) TEM, (B) HRTEM, and (C) EDS mappings images of L-Au-ZIF.


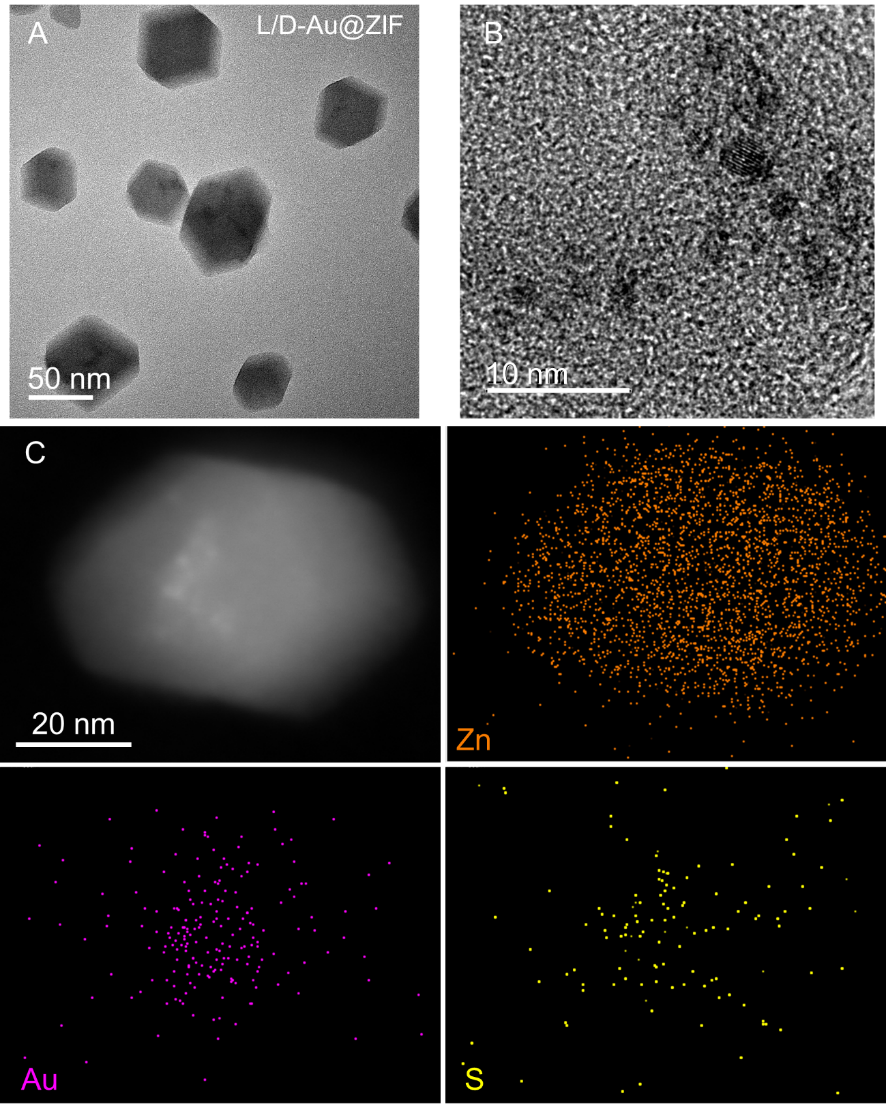


**Figure S9.** (A) TEM, (B) HRTEM, and (C) EDS mappings images of L/D-Au@ZIF.


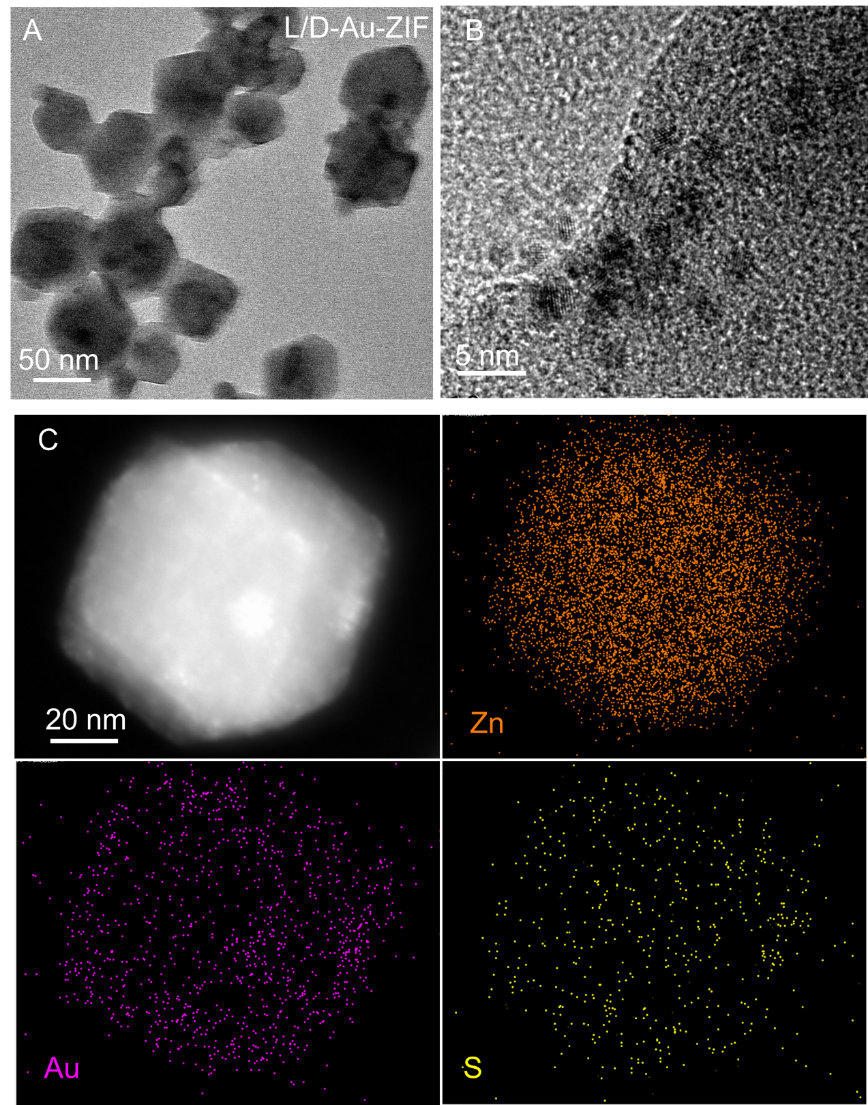


**Figure S10.** (A) TEM, (B) HRTEM, and (C) EDS mappings images of L/D-Au-ZIF.


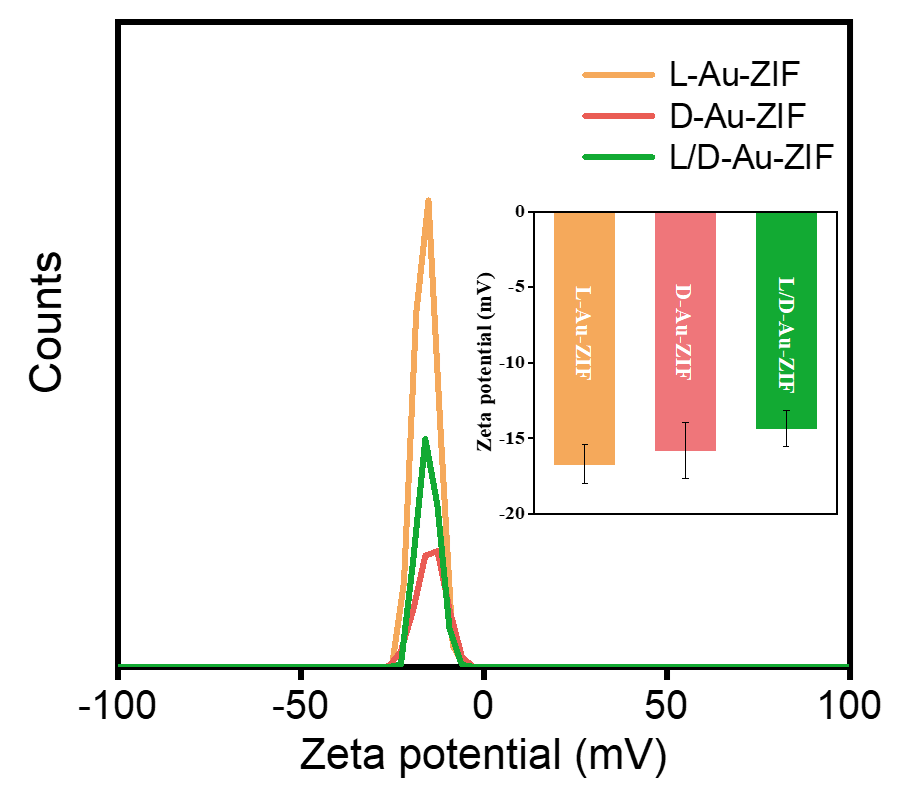


**Figure S11.** The zeta potential of L-Au-ZIF, D-Au-ZIF, and L/D-Au-ZIF.


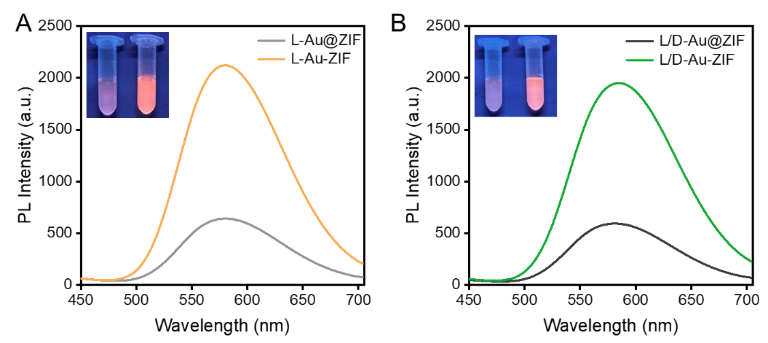


**Figure S12.** Fluorescence emission spectra of (A) L-Au@ZIF and L-Au-ZIF, (B) L/D-Au@ZIF and L/D-Au-ZIF. Inset displayed corresponding photos under UV lamp.


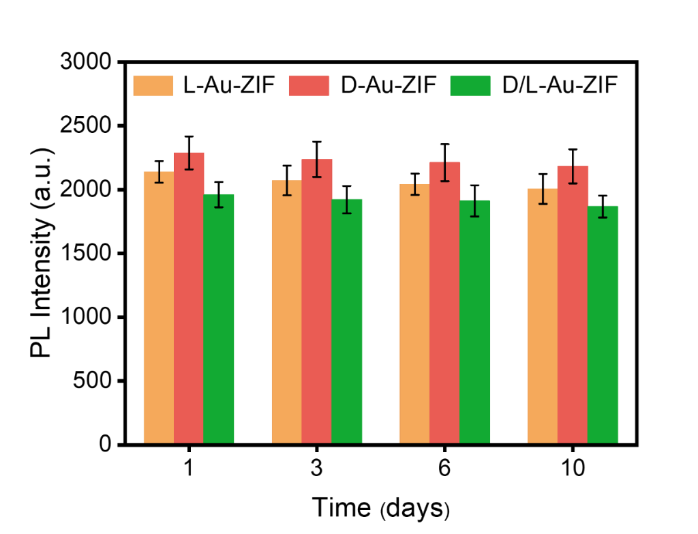


**Figure S13.** The storage stability of L-Au-ZIF, D-Au-ZIF, and L/D-Au-ZIF, n = 3 independent experiments. Data represent the mean ± SD.


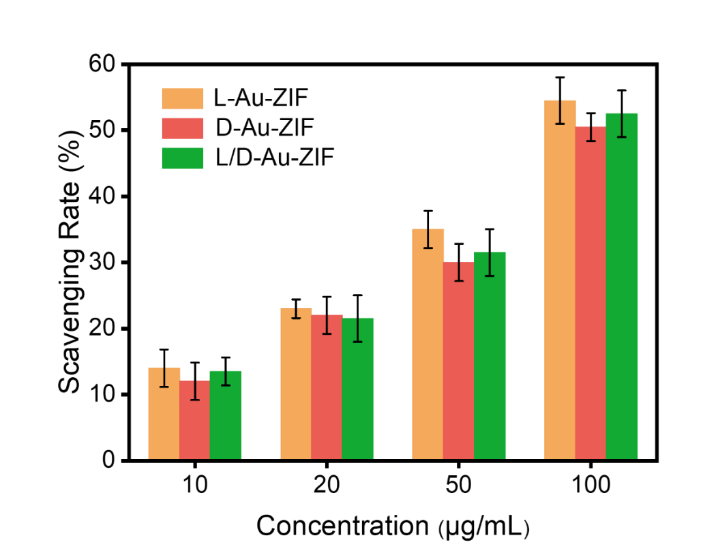


**Figure S14.** The SOD-like activities of L-Au-ZIF, D-Au-ZIF, and L/D-Au-ZIF, n = 3 independent experiments. Data represent the mean ± SD.


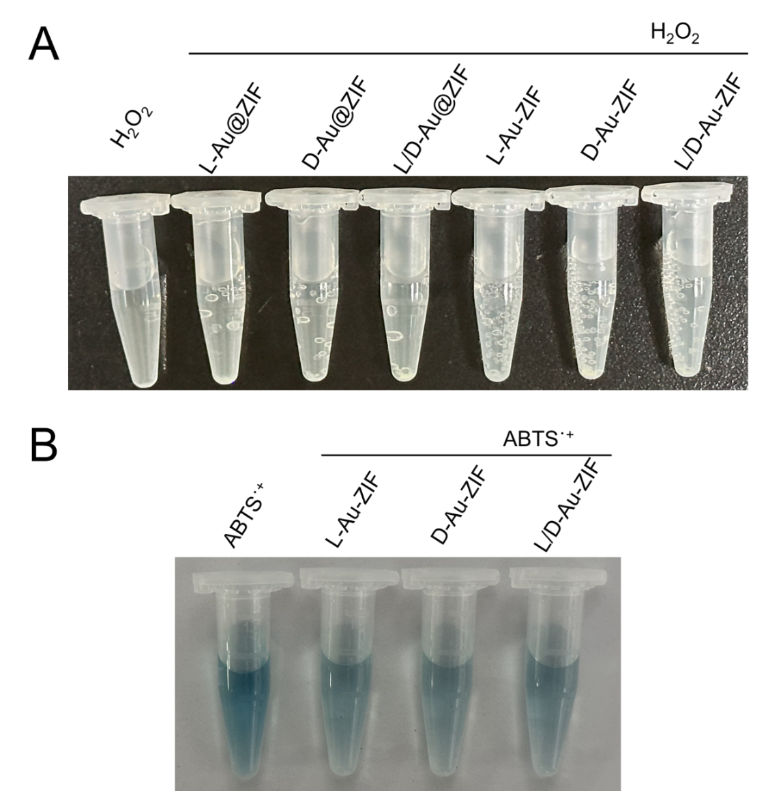


**Figure S15.**The analyses of enzymatic activities. (A) Representative O_2_ generation images of H_2_O_2_ catalyzed by L-Au@ZIF, D-Au@ZIF, L/D-Au@ZIF, L-Au-ZIF, D-Au-ZIF, and L/D-Au-ZIF for 30 mins. (B) Representative images of ABTS•+ system catalyzed by P L-Au-ZIF, D-Au-ZIF, and L/D-Au-ZIF for 15 mins.


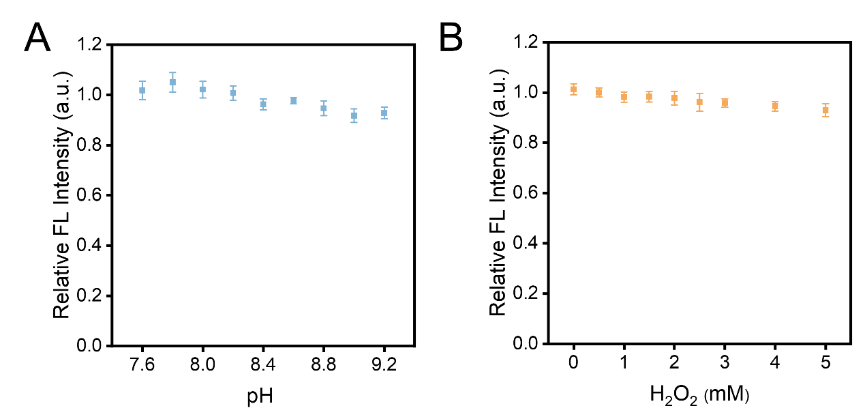


**Figure S16.** The stability of D-Au-ZIF under different pH or oxidative stress conditions.


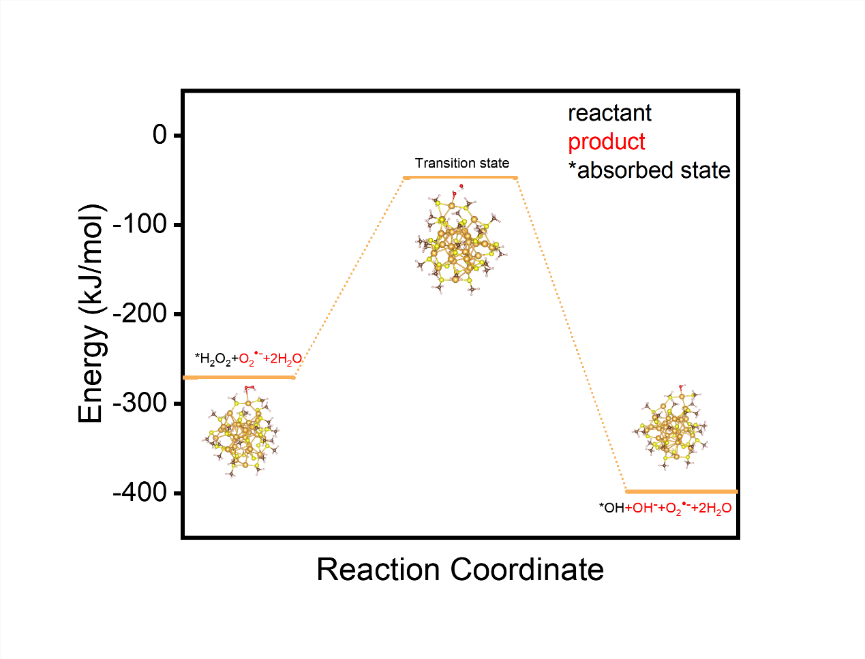


**Figure S17.** Energy proﬁles and geometry structures of the intermediate states.


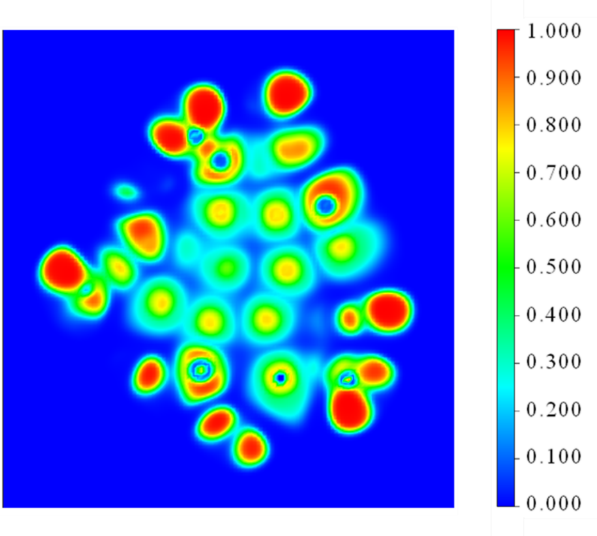


**Figure S18.**The ELF images of Au nanoclusters.


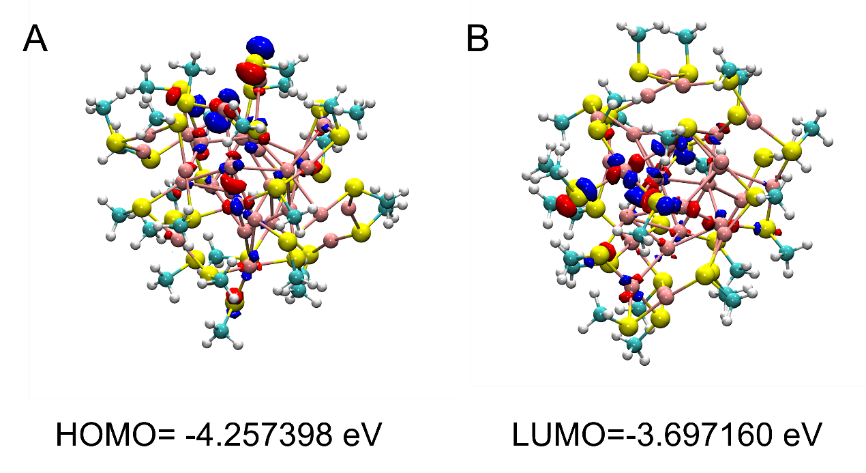


**Figure S19.** Energy alignment of the highest occupied molecular orbital (HOMO) energies and lowest unoccupied molecular orbital (LUMO) energies of Au_29_ nanoclusters.


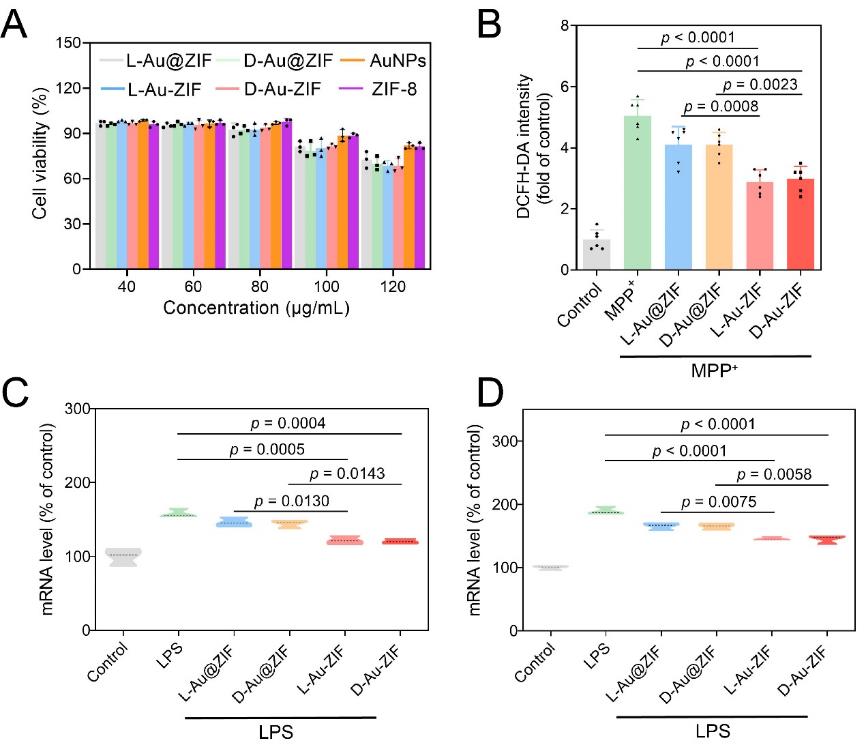
**Figure S20.** (A) Cell viability of BV2 cells exposed to NPs under various concentrations, n = 3. (B) Quantitative results of DCFH-DA fluorescence intensity, n = 6. The expression levels of proinflammatory cytokines (C) IL-6 and (D) IL-1β detected by qPCR, n = 3. Data represent the mean ± SD. The statistical analyses were conducted using GraphPad Prism 8.0.2.


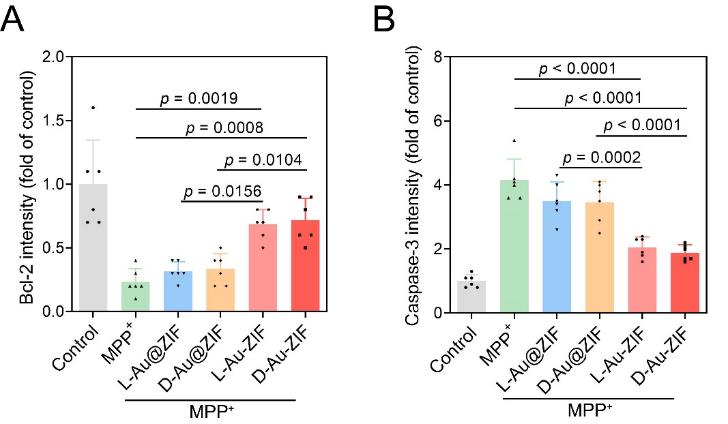


**Figure S21.** Fluorescence quantitative analyses of (A) bcl-2 and (B) caspase-3 in the immunofluorescence staining studies in SH-SY5Y cells, n = 6. Data represent the mean ± SD.


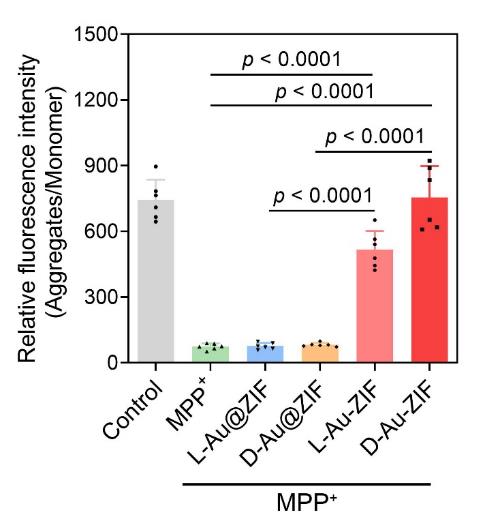


**Figure S22.** The quantitative analysis of the mitochondrial membrane potential in SH-SY5Y cells, n = 6. Data represent the mean ± SD.


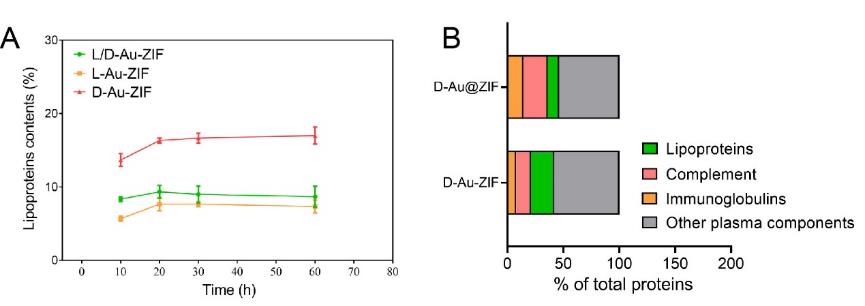


**Figure S23.** (A) The content changes in surface lipoproteins of NPs after incubation in plasma for different time. (B) The protein classifications in protein corona of D-Au@ZIF and D-Au-ZIF classified by their bioinformatic and molecular functions.


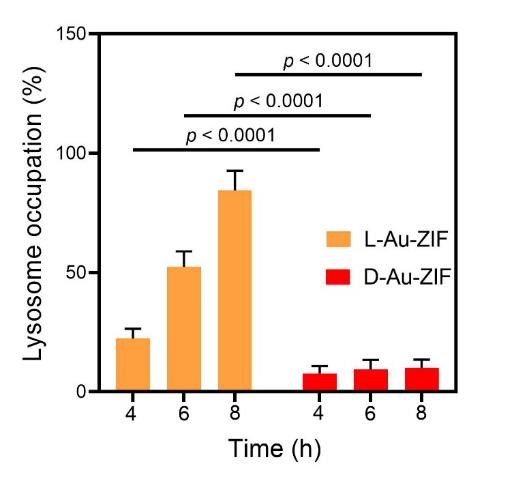


**Figure S24.** Quantitative analysis of NPs content in lysosome and total NPs after incubation of NPs for different time, n = 3. Data represent the mean ± SD.


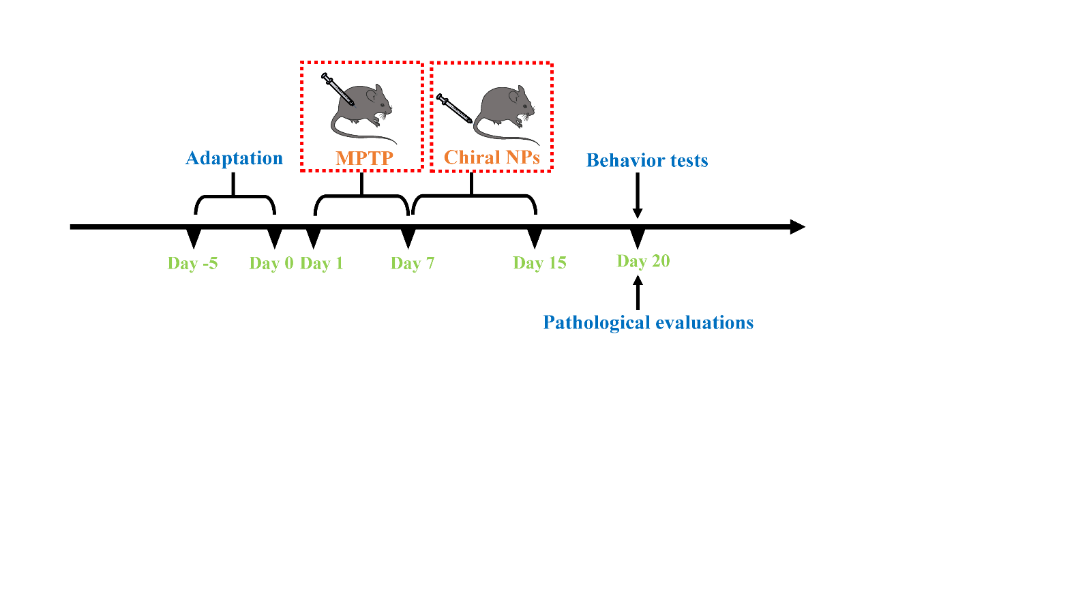
**Figure S25.** Schematic of operational flow for *in vivo* PD mouse models and chiral NPs treatment.


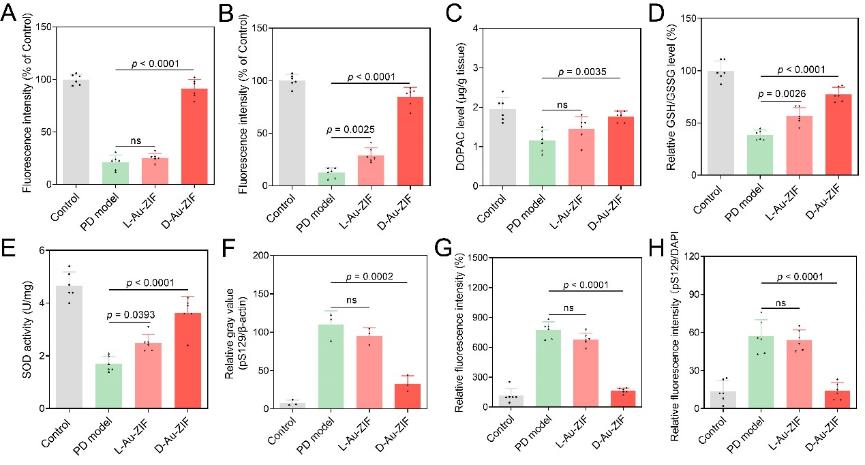


**Figure S26.** Quantitative of the relative fluorescence intensity of TH in (A) the SNpc and (B) ST region of mice brain detected by immunofluorescence staining, n = 6. Data represent the mean ± SD. (C) DOPAC levels in the striatum of PD mice after treatment, n = 6. Data represent the mean ± SD. Measurement of antioxidant capacity in the brain of PD mice based on levels of (D) GSH/GSSG, and (E) SOD activity, n = 6. Data represent the mean ± SD. (F) The expression of pS129 quantitatively analyzed in western blotting assay, n = 3. Data represent the mean ± SD. (G) Quantitative analysis of pS129 relative fluorescence intensity and (H) pS129/DAPI in immunofluorescence assay, n = 6. Data represent the mean ± SD.


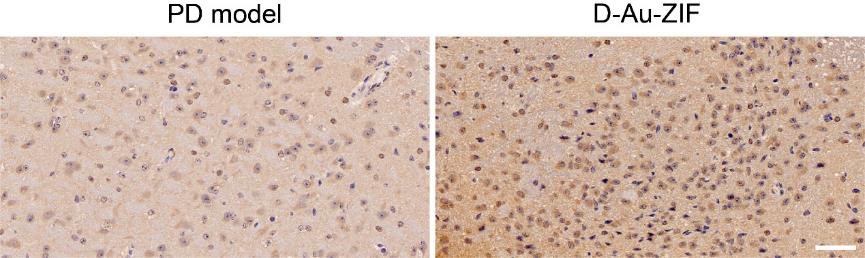


**Figure S27.** Immunohistochemical analysis of PAX6 for analyzing the role of D-Au-ZIF in promoting neurogenesis in PD models. The scale bar is 50 μm.


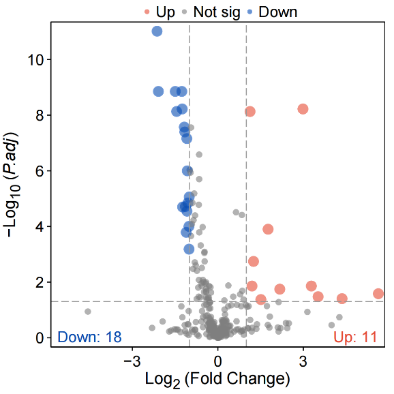
**Figure S28.** Volcano plots results showing the identified differentially metabolites after treatment with D-Au-ZIF.


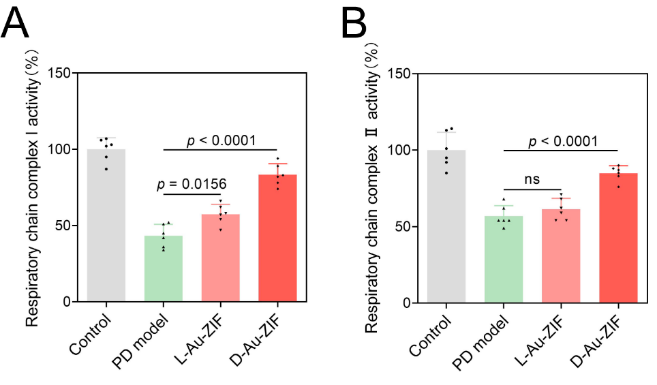


**Figure S29.** (A) Respiratory chain complex I and (B) respiratory chain complex II activity of mitochondria after chiral NPs treatment, n = 6. Data represent the mean ± SD.


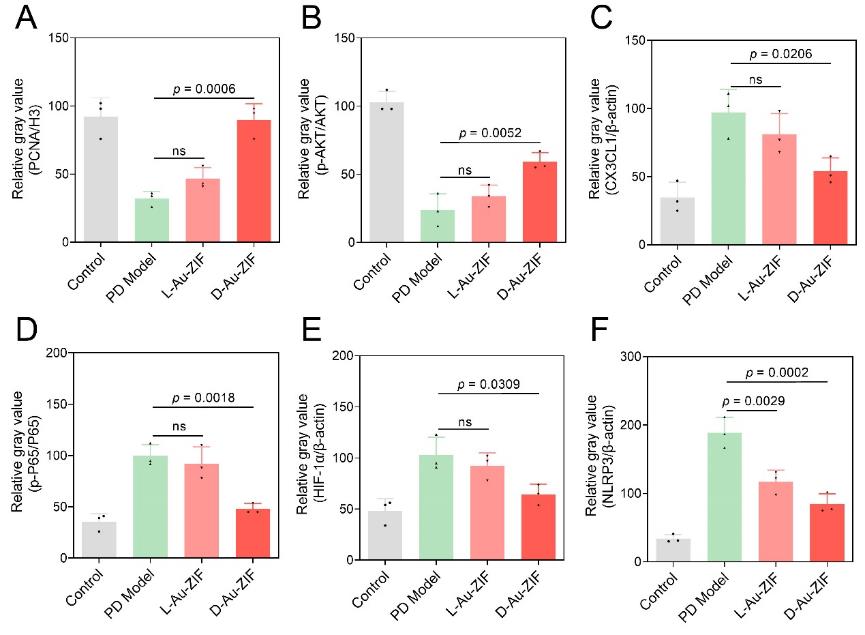


**Figure S30.** Quantitative analysis of protein expression in western blotting assays, including (A) PCNA, (B) p-AKT, (C) CX3CL1, (D) p-P65, (E) HIF-1α, and (F) NLRP3, n = 3. Data represent the mean ± SD.


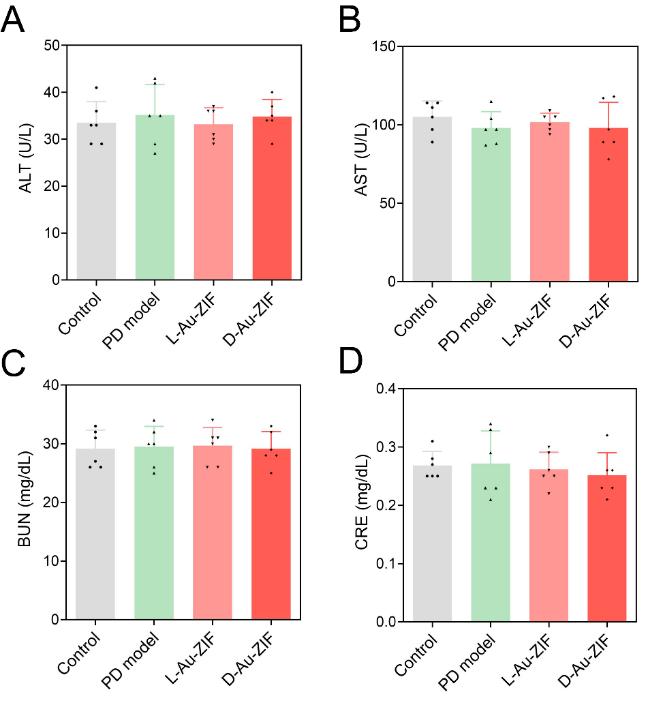


**Figure S31.** Serum biochemical analyses in PD mouse models treated with chiral NPs. (A) ALT, alanine transaminase; (B) AST, aspartate transaminase; (C) BUN, blood urea nitrogen; (D) CRE, creatinine, n = 6. Data represent the mean ± SD.


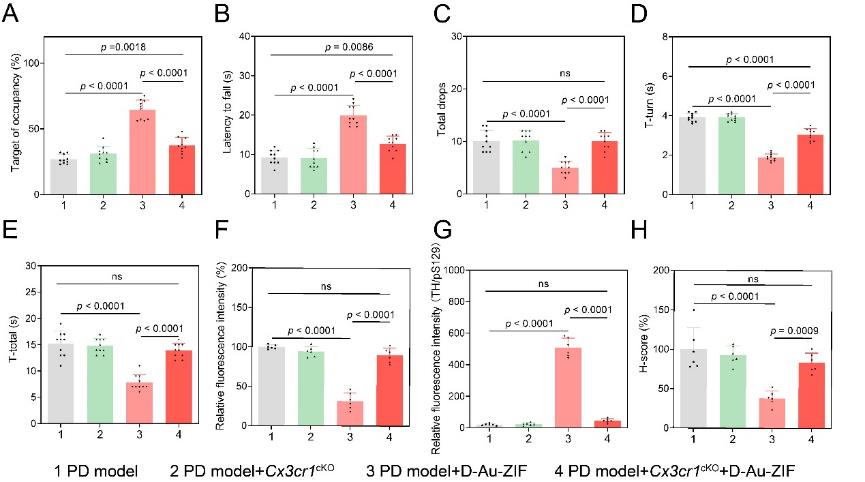


**Figure S32**. (A) The time spent in the target quadrant under various treatments in the Morris water maze test. The main indicators in the rotatory-rod test including (B) latency to fall and (C) total drops. (D) T-turn and (E) T-total in the pole-climbing test, n = 10. Data represent the mean ± SD. (F) Quantitative analysis of pS129 fluorescence in the SNpc of mice brain. (G) Quantitative analysis of TH/pS129 in the SNpc of mice brain. (H) Quantitative analysis of CD206 areas in the immunohistochemical experiment and expressed as the percentage of H-score of other groups to the PD model group, n = 6. Data represent the mean ± SD.


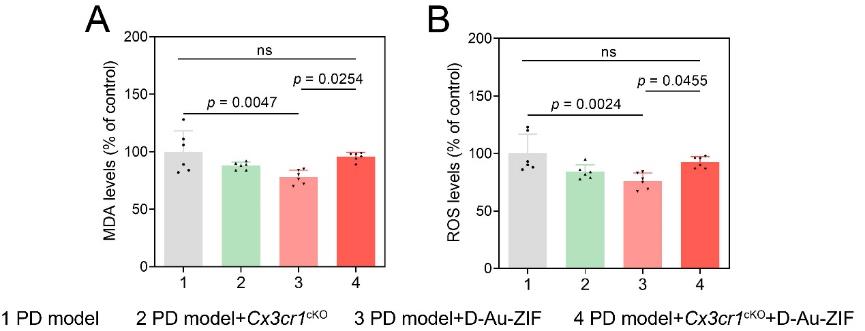


**Figure S33.** Quantitative analysis of the levels of key factors including (A) MDA, (B) ROS related to oxidative damage and mitochondrial energy metabolism processes, n = 6. Data represent the mean ± SD.

**References:**

[1] H. Tang, Q. Li, W. Yan, X. Jiang, Reversing the chirality of surface ligands can improve the biosafety and pharmacokinetics of cationic gold nanoclusters, Angew Chem Int Ed Engl (2021) 60, 13829-13834.

[2] C. Lu, L. Xue, K. Luo, Y. Liu, J. Lai, X. Yao, Y. Xue, W. Huo, C. Meng, D. Xia, X. Gao, Q. Yuan, K. Cao, Colon-accumulated gold nanoclusters alleviate intestinal inflammation and prevent secondary colorectal carcinogenesis via Nrf2-dependent macrophage reprogramming, ACS Nano (2023) 17, 18421-18432.
